# Plakophilin-2 is required for transcription of genes that control calcium cycling and cardiac rhythm

**DOI:** 10.1038/s41467-017-00127-0

**Published:** 2017-07-24

**Authors:** Marina Cerrone, Jerome Montnach, Xianming Lin, Yan-Ting Zhao, Mingliang Zhang, Esperanza Agullo-Pascual, Alejandra Leo-Macias, Francisco J. Alvarado, Igor Dolgalev, Thomas V. Karathanos, Kabir Malkani, Chantal J.M. Van Opbergen, Joanne J.A. van Bavel, Hua-Qian Yang, Carolina Vasquez, David Tester, Steven Fowler, Fengxia Liang, Eli Rothenberg, Adriana Heguy, Gregory E. Morley, William A. Coetzee, Natalia A. Trayanova, Michael J. Ackerman, Toon A.B. van Veen, Hector H. Valdivia, Mario Delmar

**Affiliations:** 1 Leon H Charney Division of Cardiology, NYU School of Medicine, 520 First Avenue, New York, NY 10016 USA; 2 Center for Arrhythmia Research, Division of Cardiology, University of Michigan, 2800 Plymouth Road, Ann Arbor, MI 48109 USA; 3 Department of Molecular and Integrative Physiology, University of Michigan, 2800 Plymouth Road, Ann Arbor, MI 48109 USA; 4 Genome Technology Center, NYU School of Medicine, 520 First Avenue, New York, NY 10016 USA; 5 Institute for Computational Medicine and Department of Biomedical Engineering, Johns Hopkins University, 3400N Charles St., Baltimore, MD 21218 USA; 6 Department of Medical Physiology Division of Heart & Lungs University Medical Centre Utrecht, Yalelaan 50, 3584CM, Utrecht, The Netherlands; 7 Department of Pediatrics, NYU School of Medicine, 520 First Avenue, New York, NY 10016 USA; 8 Departments of Cardiovascular Diseases/Division of Heart Rhythm Services, Windland Smith Rice Sudden Death Genomics Laboratory, Mayo Clinic, 200 First Street SW, Rochester, MN 55905 USA; 9 Department of Pediatric and Adolescent Medicine/Division of Pediatric Cardiology, Windland Smith Rice Sudden Death Genomics Laboratory, Mayo Clinic, 200 First Street SW, Rochester, MN 55905 USA; 10 Department of Molecular Pharmacology and Experimental Therapeutics, Windland Smith Rice Sudden Death Genomics Laboratory, Mayo Clinic, 200 First Street SW, Rochester, MN 55905 USA; 11 Department of Cell Biology and Microscopy Core, NYU School of Medicine, 520 First Avenue, New York, NY 10016 USA; 12 Department of Biochemistry and Molecular Pharmacology, NYU School of Medicine, 520 First Avenue, New York, NY 10016 USA; 13 Departments of Pediatrics, Physiology & Neuroscience and Biochemistry and Molecular Pharmacology, NYU School of Medicine, 520 First Avenue, New York, NY 10016 USA

## Abstract

Plakophilin-2 (PKP2) is a component of the desmosome and known for its role in cell–cell adhesion. Mutations in human PKP2 associate with a life-threatening arrhythmogenic cardiomyopathy, often of right ventricular predominance. Here, we use a range of state-of-the-art methods and a cardiomyocyte-specific, tamoxifen-activated, PKP2 knockout mouse to demonstrate that in addition to its role in cell adhesion, PKP2 is necessary to maintain transcription of genes that control intracellular calcium cycling. Lack of PKP2 reduces expression of *Ryr2* (coding for Ryanodine Receptor 2), *Ank2* (coding for Ankyrin-B), *Cacna1c* (coding for Ca_V_1.2) and *Trdn* (coding for triadin), and protein levels of calsequestrin-2 (Casq2). These factors combined lead to disruption of intracellular calcium homeostasis and isoproterenol-induced arrhythmias that are prevented by flecainide treatment. We propose a previously unrecognized arrhythmogenic mechanism related to PKP2 expression and suggest that mutations in PKP2 in humans may cause life-threatening arrhythmias even in the absence of structural disease.

## Abstract (web summary)

It is believed that mutations in desmosomal adhesion complex protein plakophilin 2 (PKP2) cause arrhythmia due to loss of cell-cell communication. Here the authors show that PKP2 controls the expression of proteins involved in calcium cycling in adult mouse hearts, and that lack of PKP2 can cause arrhythmia in a structurally normal heart.

## Introduction

Arrhythmogenic right ventricular cardiomyopathy (ARVC), also known as “Arrhythmogenic Right Ventricular Dysplasia” (ARVD) and most recently as “Arrhythmogenic Cardiomyopathy” (ACM) is an inherited heart disease characterized by a fibrous or fibrofatty infiltration of the heart muscle, commonly—though not exclusively—of right ventricular (RV) predominance, ventricular arrhythmias and increased propensity for sudden death in the young^1^. Sudden unexpected cardiac arrest is associated frequently with exercise, most often occurs in early adulthood during the subclinical (or “concealed”) phase of the disease when overt cardiomyopathy is not yet detectable by imaging (echocardiography or cardiac MRI)^2, 3^, and is the first disease manifestation in a high proportion of probands^1, 4, 5^. Understanding electrical remodeling in the early stage of the disease is therefore paramount to understand sudden death mechanisms.

ARVC associates primarily with mutations in genes coding for desmosomal proteins. Among these, one of the most commmonly disrupted is *PKP2*, which encodes the protein plakophilin-2 (PKP2)^1–5^. Despite clear evidence that mutations in this molecule can cause ARVC in humans, fundamental knowledge related to the biology of PKP2 in adult cardiac myocytes is quite limited. Indeed, PKP2 expresses both in myocytes and in non-myocytes^6^, and is present both in cardiac progenitors and in differentiated myocytes^6, 7^. Recent studies focused on defining the contribution of non-myocyte cells to the origins of ARVC^6^; and others suggested that absence of desmosomal genes in progenitors of the second heart field during development is an important component of disease pathogenesis^8–10^. Yet, even though PKP2 is expressed abundantly in adult myocytes and likely is fundamental to function, the consequences of PKP2 deficiency specifically in myocytes, and after the heart is developed fully, remain poorly explored.

In addition to its role in cell adhesion, PKP2 is a component of the connexome^11^ and as such, scaffolds an intracellular signaling node coupled to multiple molecular pathways; dysfunction of this node leads to an expanding chain of events that disrupts the cardiac transcriptional program^7, 12^. The latter has been documented for cell systems, but evidence collected from adult PKP2-deficient hearts remains lacking. Furthermore, while PKP2-dependent changes in the transcriptome have been studied in the context of fibrogenesis and adipogenesis^7, 12^, the possibility that transcriptional modifications specifically contribute to an arrhythmogenic phenotype has not been studied.

We generated a novel cardiomyocyte-specific, tamoxifen-activated, PKP2 knockout murine line (αMHC-Cre-ER(T2)/*Pkp2* fl/fl; referred to as “PKP2-cKO”), which allowed us to control the onset of PKP2 loss of expression, limit it to adult myocytes, and establish a time line for progression of molecular and functional events. Through a combination of multiple experimental approaches including super-resolution imaging^13^ we show that PKP2 deficiency in adult ventricular myocytes is sufficient to cause an arrhythmogenic cardiomyopathy of RV predominance in mice. RNAseq data show a complex downregulation of multiple networks. Of relevance to arrhythmogenesis, molecular, functional and structural studies show the downregulation of a transcriptional network that controls calcium cycling, leading to a disruption of intracellular calcium homeostasis and a high propensity to isoproterenol- (ISO) induced arrhythmias that are prevented by flecainide treatment. Because of the intrinsic limitations inherent to all animal models, these results cannot be directly transported to human patients affected with ARVC. Yet, they imply possible new avenues of investigation in humans, namely to examine the role of intracellular calcium homeostasis as an arrhythmia trigger and as a therapeutic target in patients with mutations in PKP2, even in the absence of overt structural disease.

## Results

### Structural and electrical phenotype of PKP2-cKO mice

**Fig. 1:**
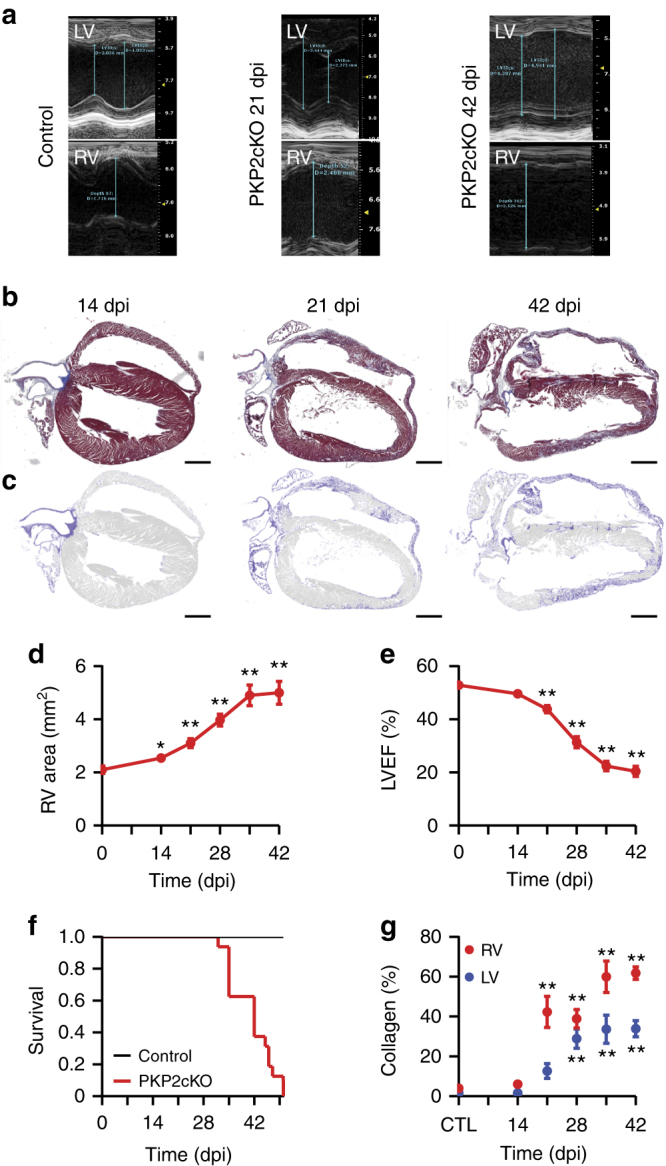
Progression of cardiomyopathy in PKP2-cKO mice. **a** Representative images of M-mode echocardiography recorded from left ventricle and right ventricle (LV; RV) of PKP2-cKO mice before (control; *left*), 21 dpi (*middle*) and 42 dpi (*right*). Notice scales on the right of each panel. **b** Masson’s trichrome staining of longitudinal heart sections of PKP2-cKO hearts at 14, 21, and 42 dpi (left, middle and right panels, respectively). **c** High contrast mask of the same sections emphasizing collagen deposition in the RV and LV in *blue*. Scale bar = 1 mm for all images. **d** Time course of change in RV area (measured by modified long axis B-mode echocardiography) in PKP2-cKO mice as a function of days after tamoxifen injection (dpi). Number of animals: *n* = 21 (0 dpi); *n* = 12 (14 dpi); *n* = 18 (21 dpi), *n* = 16 (28 dpi); *n* = 11 (35 dpi); *n* = 9 (42 dpi). **e** LV ejection fraction (LVEF) in PKP2-cKO, measured by long axis B-mode echocardiography. Number of animals: *n* = 21 (0 dpi), *n* = 13 (14 dpi), *n* = 19 (21 dpi), *n* = 16 (28 dpi), *n* = 15 (35 dpi), and *n* = 10 (42 dpi). For **d**, **e**, we obtained repeated measures from the same animals; statistical significance was calculated by paired Student’s *t*-test, comparing each value against its own control. **f** Kaplan–Meier survival curve of PKP2-cKO and control mice as a function of days after tamoxifen injection. A total of 16 PKP2-cKO and 10 control animals were followed. **g** Quantification of collagen deposition in the right (RV, *red dots*) and in the left (LV, *blue dots*) ventricle of control (CTL, *n* = 7) and PKP2-cKO hearts at 14 dpi (*n* = 5), 21 dpi (*n* = 10), 28 dpi (*n* = 8), 35 dpi (*n* = 8) and 42 dpi (*n* = 9). Statistical significance by one way ANOVA, RV and LV compared independently against corresponding control. For **d**, **e**, **g**: *p < 0.05, **p < 0.001

We generated a *Pkp2* fl/fl mice (Supplementary Fig. 1a) and crossed it with an *αMyHC-Cre-ER*(*T2*) line^13^. We refer to this line as “PKP2-cKO”. *Pkp2* fl/fl Cre-negative littermates were used as controls. Mice developed normally without functional or structural deficits. Injection of tamoxifen in Cre+ animals caused loss of PKP2 expression (Supplementary Fig. 1b–d). Using echocardiography we observed a progression from a normal heart (Fig. 1a, *left*; Control) to a cardiomyopathy of RV predominance (Fig. 1a, *middle*; 21 days post injection or “dpi”) to biventricular dilated cardiomyopathy and heart failure (Fig. 1a, *right*; 42 dpi; days counted from first day of tamoxifen injection). Histological analysis confirmed the echocardiographic findings (see four-chamber sections in Fig. 1b, c). Echocardiograms were obtained from the same group of animals at various time points for paired statistical comparisons. As shown in Fig. 1d, the first significant change was an increase in RV area at 14 dpi. Left ventricular ejection fraction (LVEF) showed a minor decrease at 21 dpi, though to levels compatible with normal function (non-failing; Fig. 1e). Heart function continued to deteriorate with time, with LVEF reaching values below 40% in all animals studied at 28 dpi and thereafter. Consistent with the impaired cardiac function, a Kaplan–Meier survival curve showed a 100% survival until day 28 and then a sharp decrease after 35 days, with only one animal reaching 50 dpi (Fig. 1f). Other echocardiographic parameters, and data for control animals, are presented in Supplementary Table 1a, b.

Histological analysis (Trichrome staining) revealed no significant increase in collagen abundance in either left or RV free wall at 14 dpi compared to controls (Fig. 1c, g). Hearts observed at 28 dpi and thereafter showed bi-ventricular dilated cardiomyopathy with extensive fibrosis in both ventricles (right panel of Fig. 1c, g). Interestingly, at 21 dpi we observed a cardiomyopathy of RV predominance (middle panel of Fig. 1c, g). While collagen occupied <20% of the area of the left ventricle free wall in all 10 animals, 5 of 10 samples showed more than 40% collagen occupancy of the RV free wall (Fig. 1g). Control mice (also injected with Tamoxifen) showed no signs of cardiomyopathy for the entire follow-up (16 weeks post injection). In summary, the combined echocardiographic and histological data show that RV dysfunction was the first manifestation of disease (at 14 dpi), followed (and not preceded) by fibrosis of RV predominance and subsequently, biventricular dilated cardiomyopathy and end-stage failure.

**Fig. 2:**
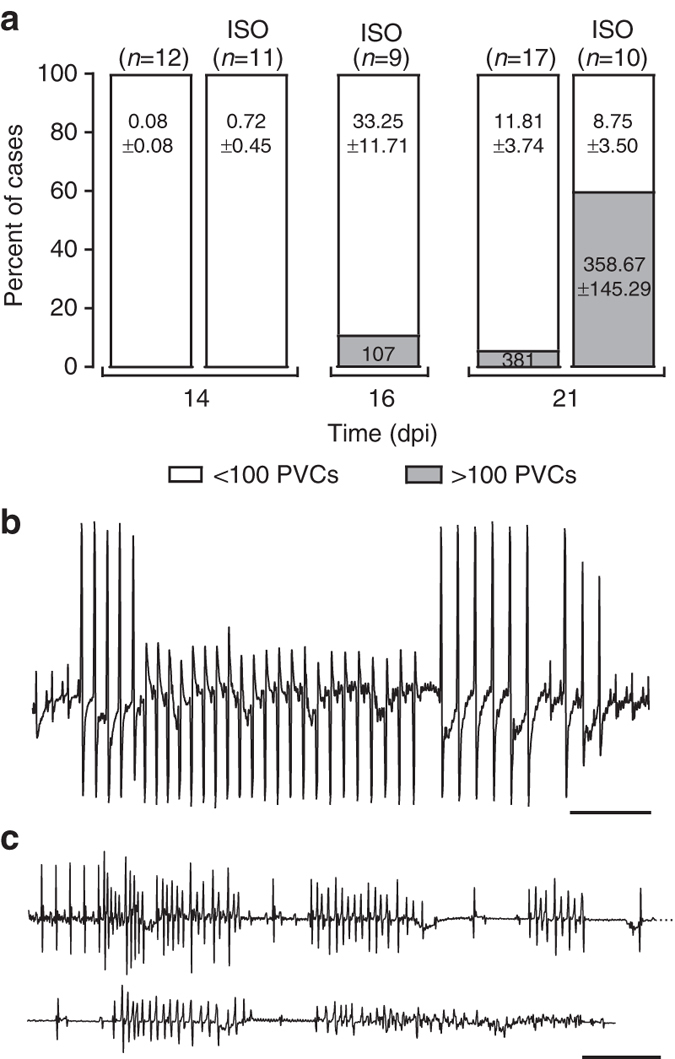
Isoproterenol-induced arrhythmias in PKP2-cKO hearts. **a** Incidence of spontaneous, and of isoproterenol-induced (ISO) PVCs during 20 min of recording in anesthetized PKP2-cKO mice as a function of days post-tamoxifen injection (dpi). Data reported as percent of total animals studied per time point and condition; number of animals in parenthesis at *top* of each bar. Numbers inside bars indicate mean ± SEM of ventricular extrasystoles. **b** Example of ISO-induced non-sustained ventricular tachycardia (NSVT) in a PKP2-cKO mouse. Scale bar = 500 ms. **c** Example of ISO-induced fatal ventricular tachycardia/fibrillation (VT/VF) in a PKP2-cKO mouse 16 days post tamoxifen injection. Scale bar = 500 ms

Electrocardiographic parameters measured in anesthetized animals were not different between groups at the time of tamoxifen injection (Supplementary Fig. 2). On follow up (same animals; repeated measures), paired comparisons showed no statistical difference in standard electrocardiographic parameters for the first 21 days post injection (Supplementary Fig. 2). Spontaneous premature ventricular contractions (PVCs) during 30 min of continuous recording were nearly absent at 14 dpi, whereas occasional extrasystoles were detected in 16 out of 17 PKP2-cKO animals at 21 dpi, with one animal showing 381 PVCs in 30 min of recording (Fig. 2a; *unlabeled bars*). The occurrence of PVCs increased further at later times. Importantly, an ISO challenge (3 mg/kg intraperitoneally (i.p.)^14^) did not increase arrhythmia burden at 14 dpi, but unveiled high arrhythmia susceptibility at 21 dpi and threafter, with 6 of 10 animals showing more than 100 PVCs in 20 min of recording, often organized in couplets, triplets and runs of non-sustained ventricular tachycardia (NSVT; Fig. 2a, *bars labeled* “ISO” and Fig. 2b). Interestingly, lethal arrhythmias were only observed at early stages, namely, mice studied at 16 dpi. Of nine mice analyzed at that time point, three died in ventricular fibrillation (VF) during ISO challenge (Fig. 2c). The transition to VF was abrupt, so the number of PVCs recorded was limited. Of note, echo images of these mice did not show structural disease. The ISO-induced polymorphic ventricular ectopy, couplets, triplets, and runs of polymorphic NSVT along with the ISO-induced VF documented lethal arrhythmias at this stage resembled a catecholaminergic polymorphic ventricular tachycardia, considering that these highly arrhythmic hearts were normal structurally at this stage.

### Differential transcriptome of PKP2-cKO hearts

**Fig. 3:**
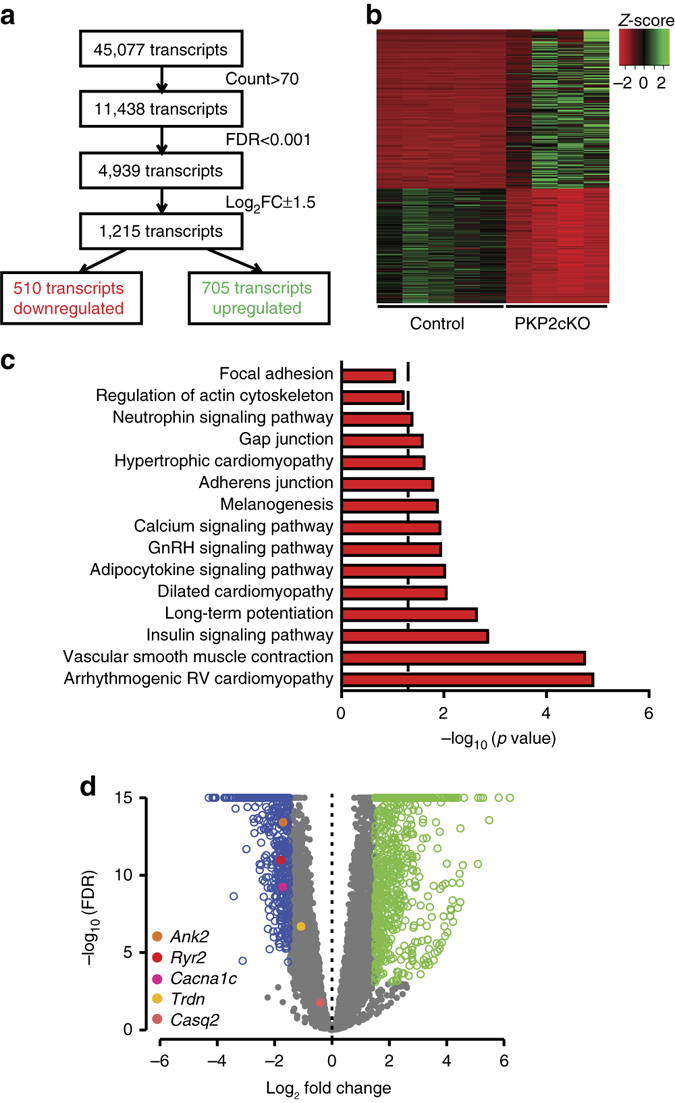
Transcriptome analysis in PKP2-cKO and control mice. **a** Flowchart of RNA-Seq analysis. **b** Heatmap of transcripts from control and PKP2-cKO hearts at 21 dpi (*n* = 5 and *n* = 4, respectively) highlighting consistency within groups. *Red* and *green*: downregulated and upregulated transcripts, respectively. **c** Significantly enriched KEGG (Kyoto Encyclopedia Genes and Genomes) categories show differentially downregulated gene pathways in PKP2-cKO hearts. **d** Volcano plot of upregulated (*green*) or downregulated (*blue*) transcripts in PKP2-cKO hearts as per inclusion criteria noted in **a**. Dots in *gray*: transcripts meeting exclusion criteria. Dots in other colors: specific transcripts noted in *bottom left* of plot

In addition to its role in cell adhesion, PKP2 scaffolds an intracellular signaling hub at the intercalated disc^12, 15^. One of the functions of this hub is to retain molecules that, if translocated in excess to the nucleus, modify transcription^7, 16^. We therefore examined the transcriptome obtained from PKP2-cKO hearts at 21 dpi in comparison to that of Cre-negative, tamoxifen injected littermate controls. Hierarchical clustering revealed a high level of homogeneity inside the groups, and a clear separation between them (Supplementary Fig. 3a). A total of 45,076 annotated transcripts from the Ensembl reference database were tested for differential expression. Stringent threshold criteria (count>70, false discovery rate, FDR < 0.001, log_2_FC±1.5) resulted in 1215 transcripts differentially expressed between PKP2-cKO and litermate controls; 510 transcripts were downregulated and 705 transcripts were upregulated in the PKP2-cKO when compared to control (Fig. 3a). Of note, for this analysis we chose hearts with no visible evidence of RV dilation. Significant differences (i.e., FDR < 0.001; log_2_FC > 1) were not observed in cardiac sarcomeric/structural genes (e.g., *Actn2*, *Obscn*, *Myh7*, *mybpc3*, *tnnt2*, *tnni3*, *actc1*, *myl2*, *myl3*, and *sptb4*) or in genes known to highly predominate in myocytes (*Gja1; Scn5a*), indicating similarity in the proportion of cardiac myocyte transcript abundance in both sample sets. A heatmap showed excellent consistency of the changes within groups (Fig. 3b).

A Kyoto Encyclopedia of Genes and Genomes (KEGG) functional network analysis of the 510 downregulated genes yielded the highest significance to the “ARVC” path; “Dilated cardiomyopathy” also scored with high significance. Pathways related to insulin signaling, adipo-cytokine interactions and gonadotropin release hormone-related cascades were prominent, emphasizing the important role that PKP2 plays in the stability of intracellular and paracrine signaling pathways, as well as in cell metabolism. Specific genes in these networks are noted in Supplementary Fig. 3b. Interestingly, the “Calcium signaling pathway” was the eighth most prominent in the list (Fig. 3c). We focused on this pathway given the high relevance of intracellular calcium homeostasis to arrhythmogenesis^17^ and our stated interest on the role of PKP2 in the control of electrical stability in the heart. As a starting point, we focused on genes known to be relevant to cardiac electrophysiology: *Ank2* (coding for ankyrin-B), *Ryr2* (coding for the cardiac ryanodine receptor), *Cacna1c* (coding for Cav1.2), and *Trdn* (coding for triadin). The positions of these genes in the volcano plot are depicted in Fig. 3d. Pathways enriched for upregulated genes involved mostly inflammatory-pro-fibrotic genes and are presented in Supplementary Fig. 3c.

### Abundance of proteins relevant to [Ca]_i_ cycling

**Fig. 4:**
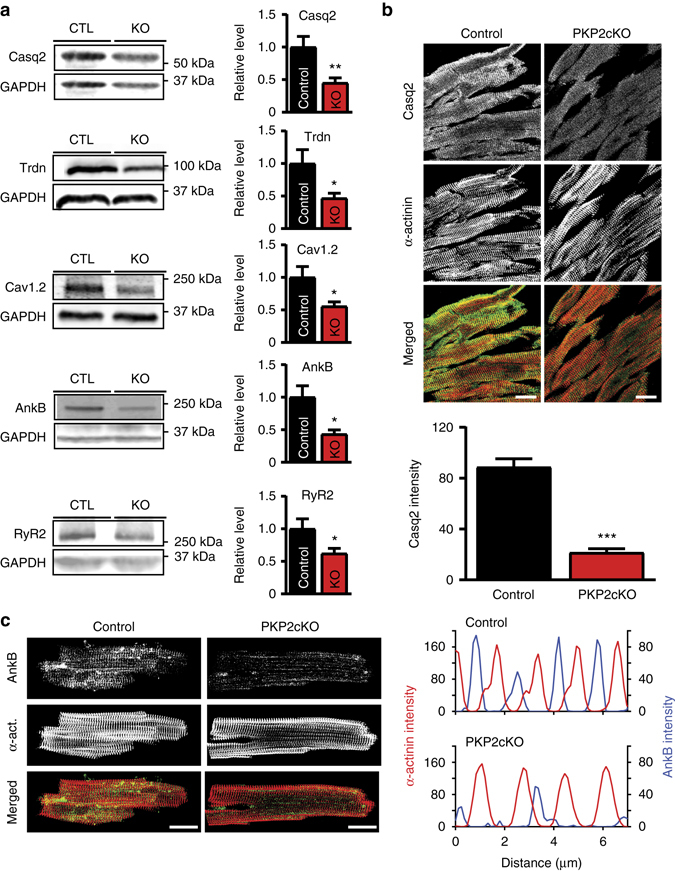
Remodeling of proteins involved in calcium signaling pathways in the PKP2-cKO mouse. **a** Representative western blots (*left*) and average densitometry (*right*; *n* = 6 for all groups) measured from control (CTL) and PKP2-cKO (KO) ventricular lysates. *p < 0.05; **p < 0.01 (Student’s *t*-test). **b** Immunofluorescence staining for Casq2 (*green*) and α-actinin (*red*) in control and PKP2-cKO ventricular sections collected at 21 dpi. Scale bar = 20 µm. Bottom panel: quantification of Casq2 intensity in PKP2-cKO vs. control samples. Analysis from 54 images, obtained from four hearts, both for PKP2cKo and control. ***p < 0.001 vs. control. **c** Immunofluorescence staining for Ankyrin B (*green*) and α-actinin (*red*) in control and PKP2-cKO heart sections at 21 dpi. Scale bar, 20 µm. Right panel: profile expression intensity of AnkB and α-actinin in PKP2-cKO and control hearts

Transcript deficits observed by RNAseq and relevant to calcium cyling were confirmed for *Ank2*, *Ryr2*, and *Trdn* (Supplementary Fig. 4). Low transcript levels correlated with decreased abundance of corresponding proteins, as demonstrated by the western blots in Fig. 4a. Furthermore, though transcript levels for *Casq2* were not significantly affected (Supplementary Table 2), protein levels were decreased (Fig. 4a), consistent with previous studies indicating that reduction in triadin leads to decreases in Casq2 protein^18^. Immunofluorescence experiments confirmed the decreased abundance of Casq2 (Fig. 4b) and AnkB (Fig. 4c). The abundance of other molecules relevant to [Ca^2+^]_i_ regulation and/or their phosphorylated forms (including RyR2), as well as the abundance of other proteins of the connexome such as Cx43, Nav1.5, and desmocollin-2 was unaffected, as seen by data in Supplementary Fig. 5. Full lanes for the western blots shown in Fig. 4 are also shown in Supplementary Fig. 5. Information about the source of the antibodies utilized, and the dilutions used for the experiments, is provided in Supplementary Table 2. Worth noting, the subdomain-specific localization of Bin-1, Cx43, and beta-catenin was preserved at this stage, indicating that changes detected in other molecules were not part of a global loss of structural integrity of the cell (Supplementary Fig. 6).

### Ultrastructural changes in the dyadic space

**Fig. 5:**
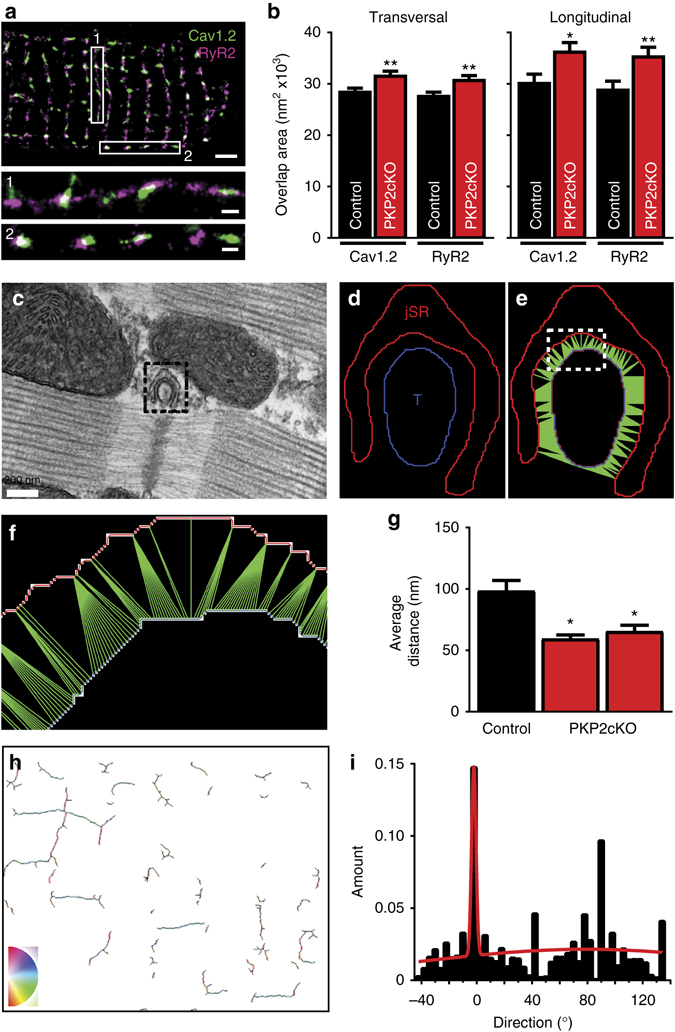
Structural changes consequent to PKP2 deletion. **a** STORM-acquired images of Cav1.2 (*green*) and RyR2 (*purple*) in a single myocyte. **b** Analysis of RyR2/Cav1.2 overlapping area in transverse (*left*) and longitudinal (*right*) clusters. *n* = 1335 and 1285 clusters from 30 control and 25 PKP2-cKO cardiomyocytes at 21 dpi, respectively. *t*-test, *p < 0.05 and **p < 0.01 vs. control. **c** 2D-EM image of PKP2-cKO ventricular tissue at 21 dpi showing a dyadic structure. Scale bar = 200 nm. **d** Boundaries of the jSR (*red*) and T-tubule (*blue*) membranes, detected from the dyad in the dotted square in **c**. **e** Acquisition of distances from each point in the T-tuble membrane to its closest neighbor in the jSR (*green lines*). **f** Close-up of the region inside the dashed square in **e**. All distances measured within a dyad were averaged to obtain the “average distance” for that dyad (expressed in nm). **g** Comparison of average data collected from one control (*n* = 30 dyads) and 2 PKP2-cKO 21 dpi samples (*n* = 41 dyads for both). Student’s *t*-test *p < 0.001 vs. control. **h** Spatial orientation of the T-tubular network, obtained from segmentation and analysis of a volume of 15 × 12 × 0.8 µm^3^ dimensions obtained by Serial Block-Face Scanning Electron Microscopy analysis. See also Supplementary Video 1 and “Methods”. The angle of the T-tubular skeleton is color-coded (*bottom left*) from −90° in *magenta* to +90° in *red*; relative to the longitudinal axis of the cell (*light blue*; 0°). **i** Histogram of orientations (from **h**), showing a strong preference for zero-degree orientation, as expected from a non-failing heart (see ref. ^60^)

Both triadin and AnkB are proteins relevant to the organization of the dyadic microdomain^19, 20^. AnkB is also relevant to calcium channel targeting^21^. We therefore speculated that changes in AnkB and triadin would alter the molecular organization of the dyad. As a first approach, we used two-color super-resolution stochastic optical reconstruction microscopy (STORM)^13, 22^ to characterize the position of Ca_V_1.2 relative to that of RyR2. To further assess data consistency, we examined clusters both from lateral membranes, and from the cell midsection. Figure 5a shows an example of images and sectors selected for analysis. Figure 5b shows that in the case of PKP2-cKO hearts, the area of overlap between Ca_V_1.2 and RyR2 clusters increased, suggesting increased proximity between the structures hosting these molecules (T-tubules and junctional sarcoplasmic reticulum (jSR), respectively).

At the ultrastructural level, T-tubules and jSR do not overlap. Yet, the limits of STORM vis a vis the dimensions of the dyadic space, combined with the fact that these are two-dimensional (2D) images of three-dimensional (3D) objects, give the impression of overlap (see, e.g., ref. ^23^) and render our measurements only an approximation of actual proximity. As a direct imaging approach with higher resolution, we measured dyadic space using transmission electron microscopy images combined with segmentation analysis^23, 24^. To obtain a more global view of the dyad we calculated the average of all distances between each point in the T-tubule and the most proximal point in the jSR (Fig. 5c–f). Though the minima of all values was not different between groups (Supplementary Table 3), we did observe significant reduction in the average of all distances separating the T-tubule from the junctional SR in PKP2-cKO hearts (Fig. 5g). Of note, using serial block face scanning electron microscopy, we found good preservation of the T-tubular network and the sarcomeric structure at this stage (Fig. 5h, i; Supplementary Movie 1), consistent with the echocardiographic finding of adequate contractile function.

### Calcium current properties depend on PKP2 expression

**Fig. 6:**
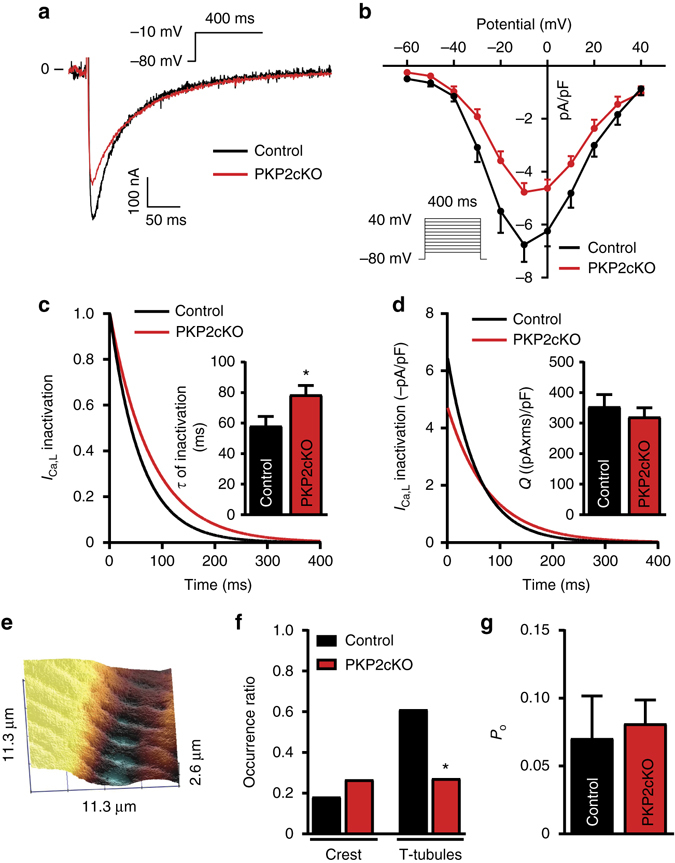
Calcium current in PKP2-cKO cardiomyocytes at 21 dpi. **a** L-type calcium current in control (*black*) and PKP2-cKO (*red*). **b** Peak L-type calcium current density as a function of voltage recorded in control (*black*) and PKP2-cKO (*red*) cardiomyocytes. Voltage clamp protocol in inset. **c** Normalized L-type calcium current decay in control (*black*) and PKP2-cKO (*red*) cardiomyocytes. Inset: Tau of inactivation of the L-type calcium current in control (*black*) and PKP2-cKO (*red*) cardiomyocytes. Student’s *t*-test, *p < 0.05 vs. control. **d** L-type calcium current decay in control (*black*) and PKP2-cKO (*red*) cardiomyocytes. Inset: Charge (*Q*) passing through calcium channels during L-type calcium current in control (*black*) and PKP2cKO (*red*) cardiomyocytes. For **b**, **c** and **d**, results collected from 13 cells, three mice in the control group and 10 cells, three mice in the PKP2-cKO group. **e** Example of SICM recording showing crest and T-tubules. **f** Occurrence ratio of calcium channels at crest and T-tubules measured by SICM in control (*n* = 11 and 18 for crest and T-tubules respectively; *black*) and PKP2-cKO (*n* = 15 and 22 for crest and T-tubules respectively; *red*) cardiomyocytes. Five mice in each group. *χ* ^2^ test, *p < 0.05 vs. control. **g** Calcium channel unitary conductance in control (*n* = 5; *black*) and PKP2-cKO (*n* = 5; *red*) cardiomyocytes. Five mice in each group

The transcriptome data indicated reduced abundance of *Cacna1c*. Consistent with this, whole-cell voltage clamp experiments revealed a decrease in average peak L-type calcium current density (Fig. 6a, b) and a slower rate of current inactivation (Fig. 6c). As a result, total charge (the integral of current over time) was the same, though the time course of its entry into the cell was different between groups (Fig. 6d). Separate scanning patch clamp studies^25^ demonstrated reduced probability of recording calcium channels at the T-tubules (Fig. 6e, f), consistent with reduced abundance of Ca_V_1.2 and reduced whole-cell current amplitude. Of note, channel open probability was not different from control (Fig. 6g) nor did we see an increased abundance of channels at the cell crest, in contrast with previous scanning patch clamp studies in failing myocytes^26^, supporting the notion that the cellular phenotype recorded was not that of a failing myocyte.

### RyR2 activity and its relation to PKP2 expression

**Fig. 7:**
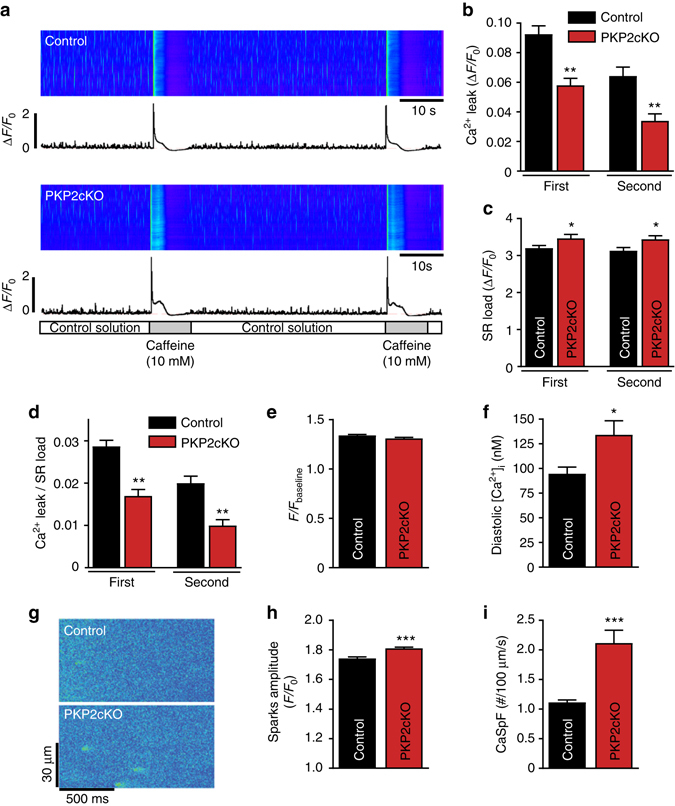
Dysruption of [Ca^2+^]_i_ homeostasis in PKP2-cKO cardiomyocytes at 21 dpi. **a** Laser scanning confocal image of Ca^2+^ in control (*top*) and PKP2-cKO (*bottom*) permeabilized cardiomyocytes. **b** Sarcoplasmic reticulum (SR) Ca^2+^ leak in control (*n* = 30 cells from three hearts, *black*) and PKP2-cKO cardiomyocytes (*n* = 23 cells from three hearts, *red*). Statistical difference was estimated by measuring the average of spontaneous Ca^2+^ release without any stimulation. **p < 0.01 vs. control. **c** SR Ca^2+^ load estimated by measuring the peak of caffeine-induced Ca^2+^ release from control (*n* = 30 cells from three hearts, *black*) and PKP2-cKO permeabilized cardiomyocytes (*n* = 23 cells from three hearts, *red*) relative to the fluorescence intensity at baseline (*F*_0_). Given that cells were permeabilized, *F*_0_ was the same for control and for PKP2-cKO. *p < 0.05 vs. control. **d** Ratio between Ca^2+^ leak and SR Ca^2+^ load in permeabilized cells, **p < 0.01 vs. control. **e** Peak of caffeine-induced Ca^2+^ release (*F*) in control (*n* = 33 cells from three hearts, *black*) and PKP2-cKO intact cardiomyocytes (*n* = 34 cells from three hearts, *red*) relative to fluorescence at baseline (*F*_baseline_). **f** Diastolic [Ca^2+^]_i_ in control (*n* = 10 cells from three hearts) and PKP2-cKO (*n* = 10 cells from three hearts) cardiomyocytes. Student’s *t*-test *p < 0.05. This result indicates that *F* at baseline (*F*_baseline_) for the data in **e** was not the same for the two groups. The combined data in **e** and **f** indicate an increase in SR load in intact cells. **g** Representative confocal line-scan images of Ca^2+^ sparks recorded in control and PKP2-cKO cardiomyocytes. **h**, **i** Bar graphs depicting Ca^2+^ spark amplitude (*F*/*F*_0_) and Ca^2+^ spark frequency, respectively, measured as the number of events per unit time and length in control (*n* = 200 sparks, *N* = 27 cells from three hearts, *black*) and PKP2-cKO intact cardiomyocytes (*n* = 457 sparks, *N* = 30 cells from four hearts, *red*). Student’s *t*-test, ***p < 0.001 vs. control

The reduced RyR2 RNA and protein abundance (Figs. 3, 4) was confirmed independently by [^3^H]ryanodine binding assays (Supplementary Fig. 7a; Supplementary Table 4), which also showed no difference in Ca^2+^-dependence of [^3^H]ryanodine binding (Supplementary Fig. 7b, c). Maximal binding (*B*_max_) normalized for RyR2 expression was not different between groups (Supplementary Table 4). Taken together, the data indicated that individual RyR2 channel activity was not altered by loss of PKP2 expression. Also consistent with decreased abundance of RyR2, analysis of Ca^2+^ sparks in permeabilized myocytes (saponin-treated; 50 µg/ml for 3–5 min) showed reduced SR Ca^2+^ leak in PKP2-cKO cells (Fig. 7a, b).

### Increased SR load and diastolic [Ca^2+^]_i_ in PKP2cKO myocytes

Caffeine pulses were delivered to permeabilized cells. The ratio of maximum fluorescence recorded upon caffeine application (*F*) over baseline fluorescence (*F*_0_) was used to assess SR Ca^2+^ load under conditions that bypass the participation of sarcolemmal channels and transporters, and that allow us to set the same baseline [Ca^2+^] for both groups. As shown in Fig. 7a, c, we detected increased SR load in PKP2-cKO cells compared to control, with a consequent decrease in leak/load ratio (Fig. 7d). Using a similar caffeine protocol but in “intact” myocytes (i.e., non-permeabilized, quiescent cells in normal Tyrode solution and exposed to a pulse of caffeine), we observed no difference in the ratio *F*/*F*_baseline_ between groups (Fig. 7e). However, separate measurements using a ratiometric dye revealed that actual diastolic [Ca^2+^]_i_ in PKP2-cKO cells was larger than control (Fig. 7f). The data in Fig. 7e, f combined lead us to conclude that, as in permeabilized cells, SR load was increased in PKP2-cKO myocytes maintained in normal Tyrode solution. Consistent with the latter, analysis of Ca^2+^ sparks in non-paced isolated myocytes revealed increased amplitude and frequency of spontaneous Ca^2+^ release (SCR) events (Fig. 7g–i).

### PKP2 loss increases excitation–contraction (e–c) gain

**Fig. 8:**
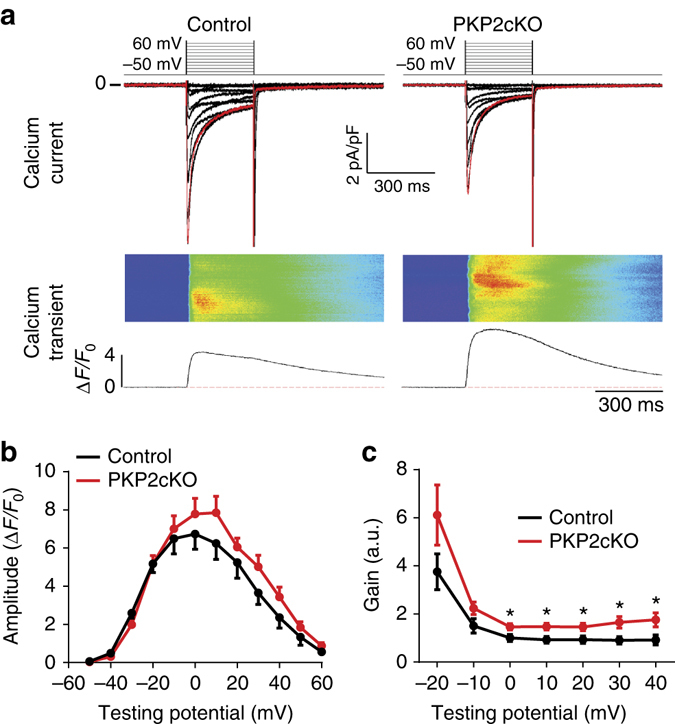
Excitation–contraction (e–c) coupling gain. **a** Representative examples of excitation–contraction coupling gain measurement in WT (*left*) and KO (*right*) cells. Cells were voltage-clamped at −50 mV, and depolarized from −40 mV to +60 mV for 300 ms in 10 mV increments. *Red traces* in top panels correspond to the current triggered by the 0 mV voltage pulse (*top*). Ca^2+^ transients were recorded simultaneously with L-type Ca^2+^ currents (*middle*). Fluorescence intensities were plotted from the Ca^2+^ images (*bottom*). **b** Amplitude of Ca^2+^_i_ transients in control and PKP2-cKO cardiomyocytes. **c** e–c coupling gain, calculated as the ratio of [Ca^2+^]_i_ transient amplitude (Δ*F*/*F*_0_) vs. *I*_CaL_ density (pA/pF), was significantly higher for PKP2-cKO cardiomyocytes, especially at positive test potentials. (Control: *n* = 10 from two mice; PKP2-cKO: *n* = 11 from three mice; *p < 0.05 vs. control by Student’s *t*-test)

The increased eagerness of the SR to release calcium in intact cells led us to examine the e–c coupling gain factor, that is, the magnitude of calcium released from the SR relative to the magnitude of *I*_Ca,L_, the Ca^2+^ current entering through L-type Ca^2+^ channels (LTCCs) upon depolarization. Myocytes were simultaneously voltage-clamped to detect *I*_Ca,L_ and imaged to detect changes in [Ca^2+^]_i_. As shown in Fig. 8a–c, the gain factor was increased significantly in PKP2-cKO cells when compared to controls. These results were reproduced by electrophysiological simulations based on the mouse ventricular model of Morotti et al.^27^ (Supplementary Fig. 8). The mathematical simulations indicated that two molecular events (reduced RyR2 and reduced Casq2) were sufficient condition for the increased amplitude of the Ca^2+^ transient, increased free Ca^2+^ in the SR, and increased e–c gain factor observed in the PKP2-cKO cells.

### Increased calcium transient amplitude and duration

**Fig. 9:**
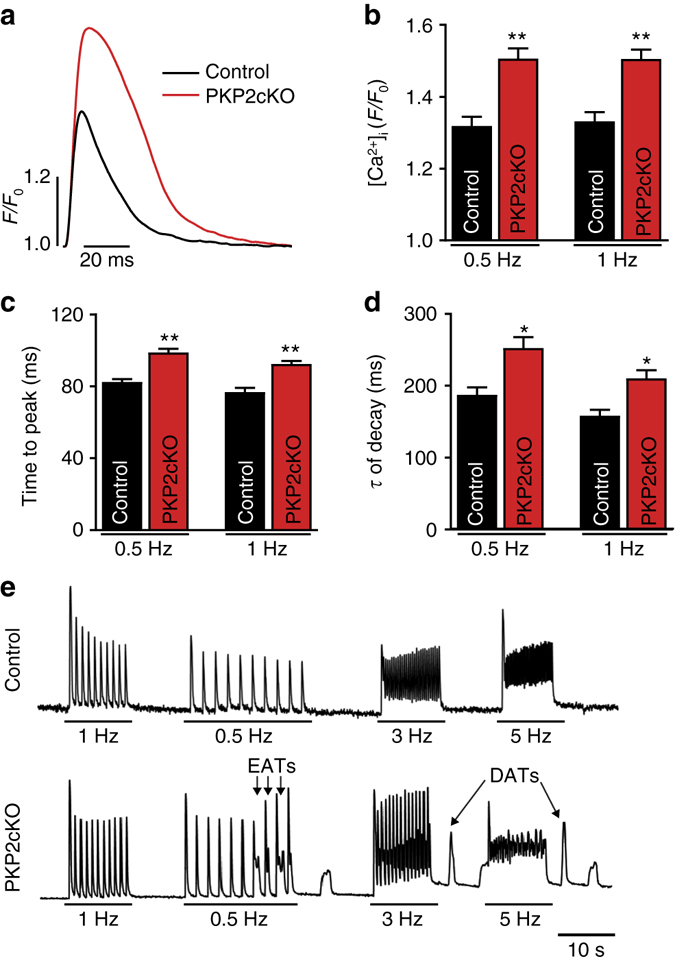
Ca^2+^ transients in PKP2-cKO cardiomyocytes at 21 dpi. **a** Ca^2+^ transients from control (*black*) and PKP2-cKO (*red*) cardiomyocytes paced at 1 Hz. **b**–**d** Quantification of time to peak (**b**), amplitude (**c**) and relaxation time constant (**d**) of calcium transients from ventricular myocytes paced at 0.5 and 1 Hz from control (*n* = 26; three mice) and PKP2-cKO (*n* = 50; three mice) cardiomyocytes. Student’s *t*-test, *p < 0.05, **p < 0.01 vs. control. **e** Examples of Ca^2+^transients in control (*top*) and PKP2-cKO cardiomyocytes (*bottom*) with different pacing rates (0.5–1–3–5 Hz). Both early after-transients (EATs) and delayed after-transients (DATs) were recorded

The results described above were consistent with an observed increase in amplitude of Ca^2+^ transients recorded from paced cells maintained in normal Tyrode solution (Figs. 8a and 9a, b). In addition, we observed prolongation of the [Ca^2+^]_i_ transient duration and increase in time to peak in PKP2-cKO cells (Fig. 9c, d) and in Langendorff-perfused hearts (Supplementary Fig. 9). We also detected a prolongation of action potential duration (APD) measured under current clamp conditions, and slight depolarization of resting membrane potential (Supplementary Fig. 10), suggesting additional involvement of repolarizing currents. Using voltage clamp, we detected no difference in the magnitude of Ni-sensitive current (ascribable to the sodium–calcium exchanger) or in the late sodium current (Supplementary Fig. 11a, b) suggesting that the difference in [Ca^2+^]_i_ transient duration may be consequent to the prolonged APD of the cells. No difference was observed for the amplitude of the inward rectifier current I_K1_ (Supplementary Fig. 11c).

### Loss of PKP2 favors early and delayed after-transients

To determine whether the molecular and functional changes observed were sufficient to generate an arrhythmogenic pattern, PKP2-cKO cells maintained in normal Tyrode solution were paced at increasing rates and [Ca^2+^]_i_ variations were monitored throughout. As shown in Fig. 9e, pacing caused spontaneous calcium release events that appeared during the time course of the elicited [Ca^2+^]_i_ transient (early after-transient) or after complete relaxation and during rest (delayed after-transient). The magnitude, temporal and spatial synchronization of the SCR events make up Ca^2+^ waves of critical mass likely sufficient to act as arrhythmogenic events, capable of generating ventricular arrhythmias.

### Effect of flecainide on ISO-induced arrhythmia burden

The experimental and numerical data indicated that loss of PKP2 expression caused excessive outflow of calcium from the jSR during each beat. Previous studies have indicated that flecainide can limit the outflow of calcium through RyR2 channels, effectively reducing the occurrence of catecholamine-triggered ventricular arrhythmias^28, 29^. We therefore tested arrhythmia burden in animals in the presence of a single dose of flecainide (40 mg/kg as in ref. ^30^) delivered i.p. The expected changes in electrocardiographic parameters resulting from flecainide exposure were observed (Supplementary Fig. 12). Furthermore, in contrast with result obtained in untreated animals, flecainide-treated PKP2-cKO mice showed absence of ISO-induced arrhythmia burden in the total recording period (20 min). These results further support the notion that arrhythmias in PKP2-cKO animals prior to cardiomyopathy may result from dysregulation of intracellular calcium cycling via increased RyR2-dependent calcium release.

## Discussion

We have generated a murine model of PKP2 deficiency targeted specifically to alpha-MHC-expressing cardiac myocytes. The CRE-ERT2 system allowed us to control the timing of *Pkp2*’s knockout. Our results show that loss of PKP2 in adult myocytes is sufficient to generate an arrhythmogenic cardiomyopathy of RV predominance in mice. We further show that a well-known effect of loss of PKP2 expression, a change in transcription program, impacts on expression of genes necessary to maintain [Ca^2+^]_i_ homeostasis and that these changes compound to facilitate a highly arrhythmogenic state. Finally, we provide evidence that treatment with flecainide, an agent that can suppress catecholaminergic polymorphic ventricular tachycardia (CPVT) caused by mutations in the *RyR2*-encoded cardiac ryanodine receptor/calcium release channel, also suppresses the ISO induced arrhythmias shown in PKP2-deficient animals. Our results, though constrained by the limitations inherent to a mouse model, imply possible new avenues of research on the pathogenesis and potential treatment of arrhythmias in patients with mutations in the gene coding for PKP2.

Previous work has suggested that the structural features of ARVC result from transcriptional dysfunction of either non-myocyte^6^ or myocyte-progenitor cells^9, 10^. Some of these studies suggested that ARVC is “a disease of cardiac stem cells”^10^. The data presented here indicate that loss of PKP2 in adult myocytes is sufficient to generate a cardiomyopathy of RV predominance, consistent with other studies suggesting that mechanical stress is a component of the cardiomyopathy. Our data, limited by the experimental model, do not discard possible changes occuring during development; it does indicate that PKP2 deficiency in adult heart can be an important contributing factor to the overall final phenotype.

The Current (2010) Task Force Criteria emphasize the presence of fibrosis, with or without adiposis, as a diagnostic criteria for ARVC^31^. Adiposis is indeed present in multiple hearts affected with ARVC, though it is considered a slowly-developing process. Our data in mice indicate that loss of PKP2 in adult myocytes does not lead to adiposis within the time frame studied, an observation consistent with other murine models of desmosomal deficiency (e.g., ref. ^32^).

Although loss of PKP2 includes both ventricles, we do observe that the RV is affected first. This suggests that RV predominance can result from the particular balance between structure and functional demand present in the RV, rather than from developmental features favoring one or the other ventricle during embryogenesis. We also observe that fibrosis occurs after mechanical dysfunction is first detected, suggesting that the fibrosis is reparative. As noted above, our data do not discard possible changes in cellular programming that occur during development affecting the control of pro-fibrotic genes in PKP2-deficient hearts; we do show that even after bypassing the developmental stage, an extensive fibrotic cardiomyopathy of RV predominance can be observed following loss of PKP2 expression.

Our study concurs with multiple others demonstrating that loss of expression of a desmosomal molecule (including PKP2) leads to transcriptional dysregulation^5, 7, 8, 12^. Our study, though, is first to define the differential transcriptome of an adult PKP2-deficient mammalian heart. While here we focused on genes controlling intracellular calcium homeostasis, other functional networks were also significantly affected and are likely to contribute to the overall functional and structural phenotype. In fact, upregulated networks included inflammatory and pro-fibrotic pathways, consistent with the anatomical changes observed at a later stage. Also of interest was the downregulation of genes involved in insulin signaling and in adipo-cytokine expression, which may play a role in the metabolic aspects of the disease^33^ as well as in the control of intracellular and paracrine signaling stability, as noted by other investigators^7, 8, 12^. Our animal model can be used for future experiments in which this particular angle can be studied in the adult heart.

The changes observed in the transcriptome included elements of the calcium signaling pathways. Whether the group of genes affected share a common transcriptional regulator (as it has been shown in other cases; see refs. ^34, 35^) that is influenced (directly or indirectly) by loss of PKP2 expression is an attractive possibility that deserves future investigation.

Our data unveiled a reduced abundance of AnkB in PKP2-cKO hearts. There are similarities between the phenotype observed in our mice and that reported for mice deficient in the AnkB protein^20^. These include increased calcium transients and SR calcium content, as well as increased frequency of calcium sparks in intact myocytes. The latter leads us to speculate that the reduced abundance of AnkB may play a critical role in the overall phenotype consequent to loss of *Pkp2* expression.

In a previous study, Chopra et al. demonstrated that loss of triadin expression caused a reduction in Casq2 abundance^18^. This may explain the significant decrease in Casq2 protein (and not transcript) observed in our studies. Since Casq2 acts as negative regulator of RyR2 openings^36^ and RyR2 density is decreased in PKP-KO hearts, the decreased Casq2 levels may reflect a compensatory effort of the cell to maintain calcium homeostasis, but at the cost of enhancing the incidence of arrhythmogenic SCR events^37^.

Chopra et al. also found that reduced Trdn altered the ultrastructure of the couplon^18^. Although the specific ultrastructural changes noted by those authors were not identical to those in our study, we note a consistency in the fact that altered scaffolding protein expression can change the anatomy of the calcium release functional unit. Overall, the emerging picture is one in which altered expression of scaffolding molecules in the dyad secondary to transcriptional dysregulation leads to altered ultrastructure of the dyadic space. The specific role that these structural changes play in the increased e–c coupling gain factor or in other features of the phenotype, remains to be defined.

Overall, our data indicate that multiple molecules relevant to calcium handling are affected by the loss of PKP2 expression. Our results show a complex molecular phenotype with a clear functional consequence: an increased susceptibility to arrhythmias that, in a whole heart, could lead to death. We acknowledge at the outset that the genetic deficit in PKP2 created for the purpose of this study does not reproduce that of humans with ARVC, where PKP2 deficiency is very rarely recessive and it does not affect only one cell type or developmental stage. (In fact, from a strict definition, we are unaware of any murine or cell system including that of induced pluripotent stem cell-derived cardiomyocytes or human induced pluripotent stem cell-derived cardiomyocytes (hIPSC-CMs) that models ARVC.) Yet, some important convergence with data obtained from human studies deserves mention. In particular, Denis et al. recently reported that an ISO challenge effectively identifies arrhythmia susceptibility in patients at a pre-clinical stage of ARVC^38^. A calcium dysregulation was also reported (although with differences in the actual phenotype) in hIPSC-CMs deficient in PKP2^33^, and a separate study indicated that flecainide may be effective for the treatment of arrhythmias in some ARVC patients^39^. Our data provide a possible mechanism (yet to be fully explored) for both the response to the ISO challenge and to the flecainide treatment in humans, and creates a foundation for future studies to explore the relation between the structural integrity of PKP2, and arrhythmias originating from dysregulation of intracellular calcium homeostasis.

**Fig. 10:**
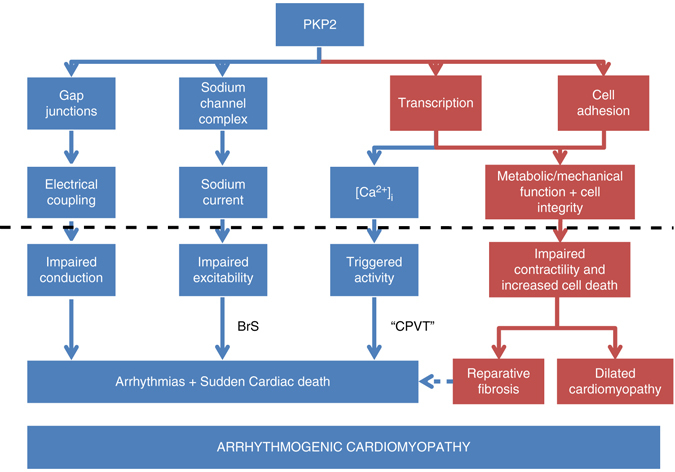
Diagrammatic representation of the function of PKP2 in the adult heart. PKP2 scaffolds a signaling node at the intercalated disc. From that position, it covers four known functions: Maintains intercellular coupling and sodium channel function, modulates transcription, and facilitates cell–cell adhesion. In the present study, we show that transcriptional regulation impacts on [Ca^2+^]_i_ homeostasis. These functions are necessary for normal electrical and mechanical function. Loss or mutations in PKP2 (signaled by the *black horizontal dotted line*) could independently impair electrical (*blue*) or mechanical (*red*) function. A predominant effect in one of these descending branches would yield either an electrical phenotype (resembling Brugada syndrome—BrS—or resembling catecholaminergic polymorphic ventricular tachycardia—CPVT), a mechanical phenotype (e.g., dilated cardiomyopathy) or a combination of both, which would yield the “classic” phenotype of ARVC

While our data are constrained by the limitations inherent to the animal model, it also implies possible new avenues of research for humans. In that context, we hypothesize that mutations in PKP2 could underlie some catecholamine-sensitive life-threatening arrhythmias in young individuals even in the absence of structural disease, thus leading to a clinical diagnosis of CPVT. The existence and prevalence of *PKP2* mutations in heretofore genotype negative CPVT remains to be determined. Overall, we speculate that as a protein that scaffolds multiple complexes in the connexome and participates in separate but parallel functions, PKP2 deficiency can lead to more than one phenotype (see diagram in Fig. 10). Importantly, those phenotypes can mimic the ones classified as pertaining to other diseases, such as Brugada syndrome^40^ or CPVT. PKP2 may therefore be more than just an ARVC-susceptibility gene. Instead, *PKP2* may be a gene that can, if mutated, precipitate phenotypes that vary from purely arrhythmogenic (a “channelopathy”) to that of a severe mechanical dysfunction.

In conclusion, we have used a murine model of PKP2 deficiency to demonstrate a transcriptional dysregulation of molecules involved in intracellular calcium handling/cycling and a consequent increase in arrhythmogenesis. As in all other studies that rely on mouse models, our goal was not to model the entire disease constellation but rather, to understand the role that is played, in one specific cell type and at a given time point in development, by a molecule (PKP2) known to be responsible for a heritable disease in humans (ARVC). In doing so, we demonstrate that the loss of PKP2 only in adult myocytes is sufficient for the development of a catecholamine-inducible arrhythmogenic phenotype without overt structural disease, that later gives way to an arrhythmogenic cardiomyopathy of RV predomininace and finally, a biventricular dilated cardiomyopathy leading to end-stage heart failure. Our studies reduce the gap that separates us from understanding the biology of PKP2 in the adult heart and as such, open new avenues to investigate the causes of disease and the proper targets for future therapeutic intervention in humans.

## Methods

### Generation of cardiac-specific PKP2-cKO mice

A C57BL/6 PKP2 fl/fl mice line was generated and crossed with the αMyHC-Cre-ER(T2) line, as previously published^41^ (Supplementary Fig. 1). Two forward loxP sites were designed and introduced into the construct flanking mouse PKP2 exons 2 and 3, with a downstream neomycin selection cassette. The linearized targeting construct was electroporated into C57B/6 derived embryonic stem (ES) cells and the resultant embryonic stem (ES) cell clones were identified. The confirmed positive ES cells were injected into isogenic blastocyts, and microinjected into the foster mice. The neo cassette was excised by crossing the F1 heterozygous mice with FRT mice. The PKP2 flox/flox mice were mated to αMyHC-Cre-ER(T2) mice to obtain flox/flox/Cre+ mice which contains the α myosin heavy chain promoter and the ligand binding domain of the human estrogen receptor. The resulting mice (PKP2-cKO) developed normally without functional or structural deficits. All procedures conformed to the Guide for Care and Use of Laboratory Animals published by the US National Institutes of Health (NIH Publication no. 58-23, revised 1996) and were approved by the NYU IACUC committee under protocol number 160726-01. Mice were injected 4 consecutive days with tamoxifen (3 mg dissolved in sterile peanut oil with 10% ethanol; mice weight hovered ∼28 – 30 g, giving an approximate tamoxifen dose of 0.1 mg of tamoxifen per gram of body weight). Binding of tamoxifen to the estrogen receptor induced the cardiomyocyte specific Cre-mediated deletion of the *Pkp2* gene. All experiments were performed in C57BL/6 PKP2-cKO mice and CRE-negative, tamoxifen treated, C57BL/6 littermates were used as controls. Both genders were included. All animals were between 3 and 4 months of age.

### PCR validation of gene expression

Total RNA was extracted from the heart tissue of PKP2-cKO and control mice using RNeasy Mini Kit (QIAGEN). The cDNA was generated by reverse transcription (RT)-PCR with SuperScript VILO cDNA Synthesis Kit (Life Technologies). PCR reactions were performed using the cDNA and the primers targeting to the specific exon region in each gene. Resultant reactions were loaded on 2% agarose gel and visualized with Bio-Rad ChemiDoc.

### Electrocardiograms recordings

Mice were anesthetized with 1.5% isoflurane in 700 ml O_2_ per minute via a nose cone (following induction in a chamber containing isoflurane 4–5% in oxygen). Rectal temperature was monitored continuously and maintained at 37–38 °C using a heat pad. Three lead ECG (leads I, II, III) were recorded from sterile needle electrodes inserted subcutaneously in each forelimb and hindlimb. The signal was then acquired and analyzed using a digital acquisition and analysis system (Power Lab; AD Instruments; LabChart 7Pro software version). ECG parameters were quantified after 1–2 min from anesthesia induction, in order to stabilize the trace. QT interval was defined as the time elapsed from the beginning of the major deflection representing the QRS to the end of the secondary slow deflection, as described by Danik et al.^42^ QT intervals were corrected for RR interval by the equation (QTc = QT/(RR/100)^1/2^), according to Mitchell et al.^43^ Analysis was performed on lead II electrocardiograms. For spontaneous arrhythmias monitoring, mice were monitored for 30 min after anesthesia induction. For ISO experiments, after anesthesia induction and ECG stabilization for 1–3 min, 3 mg/kg ISO were injected i.p. in a single bolus and the ECG was recorded for 20 min post injection^14^. Flecainide experiments were performed at 21 dpi. After anesthesia induction and ECG stabilization for 1–3 min, flecainide 40 mg/kg was administered in a single bolus as previously published^30^. After 15 min, upon evidence of drug effect by ECG parameters (P wave duration, PR and QRS intervals prolongation), ISO 3 mg/kg was administered i.p. and the ECG recorded for an additional 20 min.

### Echocardiography

Transthoracic echocardiography was performed using a Vevo2100 Imaging System (VisualSonics Inc., Toronto, Canada) with a 30 MHz probe. Briefly, after induction of anesthesia in a chamber containing isoflurane 4–5% in oxygen, the mouse was positioned supine on a heat pad in order to maintain body temperature at 37–38 °C and anesthesia was maintained with 1.5% isoflurane in 700 ml O_2_ per minute via a nose-cone. Recordings were obtained in parasternal long and short axis views. Quantitative measurements were assessed offline using the Vevo2100 analytical software. A B-mode parasternal long axis view was used for LVEF and volumes measures. Left ventricular fractional shortening was calculated from the parasternal short axis view (M-mode)^44^. A B-Mode modified parasternal long axis view of the RV was used to visualize the chamber and to measure the RV diameter: during diastole, the diameter was measured half-way between the pulmonary and tricuspid valves from the free wall to the interventricular septum^45^. Pulsed-wave Doppler was used to quantify right ventricular outflow tract velocity time integral (RVOT-VTI) and this value was used as a surrogate for RV function^45^.

### Histology

Hearts were fixed with 4% paraformaldehyde in phosphate buffered saline (PBS), embedded in paraffin, and cut into 5 μm thick sections. Sections were stained with Masson’s Trichrome according to the manufacturer’s instructions. Briefly, paraffin embedded sections were incubated at 60 °C for 30 min and rehydrated. Slides were then incubated in Bouin’s solution, 60 °C for 1 h followed by wash steps and immersed in Weigert’s working hematoxylin solution for 10 min. After repeated washes, slides were immersed in Biebrich Scarlet–Acid Fuchsin solution for 5 min and phosphomolybdic acid solution for 10 min prior to putting them into Aniline blue for 5 min. After a wash, slides were incubated in 1% acetic acid for 1 min, rinsed, dehydrated and mounted with Permount Mounting Medium. Stained sections were scanned at a ×40 magnification on a Leica SCN400F Whole Slide Scanner. The ImageJ (NIH) software was used for analysis of tissue section. By defining regions of interest (ROIs), three ROIs for each ventricle were selected (base, free mid-wall, apex) and the interventricular septum was excluded. For each ROI, the area of collagen (blue staining) was normalized to the area of tissue.

### Western blot analysis

To analyze protein expression levels, PKP2-cKO and controls mice were euthanized (CO_2_ inhalation 2% for 5–10 min, confirmed by cervical dislocation) at 21 dpi and ventricular samples were cryopreserved immediately in liquid nitrogen. Ventricular samples were then homogenized in extraction buffer containing protease and phosphatase inhibitors (150 mM NaCl, 0.02% Sodium azide, 1% Triton X-100, 1 mM PMSF, 1 mM Na_3_VO_4_, 50 mM NaF, 50 mM Tris-HCl, pH 8.0 and Complete Protease Inhibitor (Roche)). Protein concentration was determined using BCA kit (Invitrogen). Samples were run on 4–12% precast polyacrylamide SDS gradient gels (Invitrogen) and transferred onto nitrocellulose membranes (Bio-Rad) subsequently incubated in blocking buffer consisting of PBS with Tween-20 (0.1%) and 1% nonfat dry milk. Membranes were then incubated with specific primary antibodies diluted in 1% nonfat dry milk overnight at 4 °C followed by wash steps and secondary antibodies (see ‘Antibodies’ section). Antigen complexes were visualized and quantified with the Odyssey Infrared Imaging System (LI-COR).

### Immunohistochemistry

PKP2-cKO and controls mice were euthanized and the hearts were excised and immediately fixed in 4% paraformaldehyde. The hearts were washed in ice-cold PBS and equilibrated in 30% sucrose at 4 °C overnight. The samples were then embedded into Tissue-Tek OCT compound (Fisher Scientific), and frozen tissues sections were cut (5 μm) and collected on Superfrost Plus microscope slides (Fisher Scientific). Sections were permeabilized in 0.1% Triton X-100 (Sigma) for 30 min and subsequently incubated in blocking buffer: 2% bovine serum albumin (Sigma), 2% glycine (Sigma), 0.2% gelatin (Sigma), for 30 min with 10% Normal Goat Serum, followed by an 1 h incubation at room temperature with primary antibodies diluted in blocking buffer blocked with 3% Normal Goat Serum. Sections were then washed in PBS and incubated with secondary antibodies with Alexa Fluor dyes (see ‘Antibodies’ section) in blocking buffer with 3% Normal Goat Serum for 30 min before mounting. Slides were coverslipped with ProLong Gold antifade mountant with DAPI (ThermoFisher). Confocal images were taken with a Leica TCS SP5 confocal microscope using Leica LAS AF acquisition software. Image analysis and quantification is made using ImageJ software (NIH).

### Antibodies

The following primary antibodies were used: monoclonal mouse anti-PKP2 (K44262M; Biodesign International, Meridian Life Sciences), monoclonal mouse anti-AnkyrinB (N105-17; BioLegend), polyclonal rabbit anti-Cav1.2 (ACC-003; Alomone), polyclonal rabbit anti-CASQ2 (PA1-913; ThermoFisher), monoclonal mouse anti-RyR2 (MA3-916; ThermoFisher), polyclonal rabbit anti-RyR2 phospho serine-2808 and anti-RyR2 phospho serine-2814 (A010-30 and A010-31 respectively; Badrilla), monoclonal mouse anti-TriadinT32 (MA3-927; ThermoFisher), polyclonal rabbit anti-NCX (sc-32881, SantaCruz), polyclonal rabbit anti-SERCA2 (PA5-29380; ThermoFisher), monoclonal mouse anti-phospholamban (sc-393990; SantaCruz), polyclonal rabbit anti-phospholamban phospho serine-16 (sc-12963; SantaCruz) and phospho threonine-17 (sc-17024; SantaCruz), GAPDH (G109A; Fitzerald) monoclonal mouse anti-Cx43 (Clone 4E6.2, Millipore), polyclonal rabbit anti-β-catenin (C2206; Sigma), monoclonal mouse anti-JPH2 (sc-37086, SantaCruz), monoclonal mouse anti-Bin1 (Clone 99D; Sigma), polyclonal rabbit anti-PKC and anti-phospho PKC (#2056 and #9375; Cell Signaling), polyclonal rabbit anti-CaMKII (PA5-22168; ThermoFisher), monoclonal mouse anti-CaMKII phospho threonine-286 (MA1-047; ThermoFisher), polyclonal rabbit Desmocollin-2 (ab72792; Abcam), polyclonal rabbit anti-Nav1.5 (S0819; Sigma). Secondary antibodies for western blotting included goat-anti rabbit IRDye 800CM or goat anti-mouse IRDye 680RD antibodies (LI-COR). Secondary antibodies for immunofluorescence: Alexa Fluor488, 568 or 647 goat anti-mouse or anti-rabbit antibodies (Invitrogen). A list of all antibodies used, their manufacturer and animal source, working concentration and application are detailed in Supplementary Table 2.

### RNASeq and KEGG analysis

RNAs for five control and four PKP2-cKO mice at 21 dpi were extracted using RNA-easy Mini kit (Qiagen). RNA-Seq library preps were made using the Illumina TruSeq RNA Library Preparation Kit v2 using 500 ng of total RNA as input, amplified by 12 cycles of PCR, and run on an Illumina 2500 (v4 chemistry), as single read 50 at the Genome Technology Center at NYUMC. Approximately 200 million reads per sample were generated. Sequencing results were demultiplexed and converted to FASTQ format using Illumina Bcl2FastQ software. Quality Control (QC) for the RNA-Seq reads was assessed using FastQC software. Next, reads were aligned to the mouse genome (build mm10/GRCm38) with Spliced Transcripts Alignment to a Reference (STAR^46^). PCR duplicates were removed using the Picard toolkit (open-source, MIT license). HTSeq package^47^ was utilized to generate counts for each gene. The read counts of each transcript were normalized to the length of the individual transcript and to the total mapped read counts in each sample and expressed in counts per millions. In order to validate the intra-group homogeneity we first performed a principal component analysis (PCA). PCA is based on two principal components PC1 and PC2^48^. The first principal component (PC1) is the direction along which the samples show the largest variation. The second principal component (PC2) is the direction uncorrelated to the first component along which the samples show the largest variation. Next, we visualized the genes with a largest variance using a hierarchical clustering heatmap. For each gene, we compared the expression levels between Control and PKP2-cKO RNAs. Gene expression differences were evaluated using Fisher’s exact test after normalizing by the total number of mapped reads in each lane. The resulting *p*-values were corrected via the Benjamini and Hochberg method. Differentially expressed genes were defined as those with log_2_ changes of at least 1.5 fold between a pair of samples at FDR of 0.001 for genes with a count above 70. Supplementary Table 2 lists all the dataset. For differentially expressed genes, we carried out functional annotation analysis using DAVID^49, 50^. Differentially expressed genes were used as input gene list, and all mouse genes that were expressed in the heart were used as the background. We looked for enrichment for genetic association with KEGG pathways. Dataset was analyzed using R software version 3 and ad hoc packages.

### Cardiomyocyte dissociation

Murine ventricular myocytes were obtained by enzymatic dissociation^51^. Mice were injected with 0.1 ml heparin (500 IU/ml i.p.) 20 min before heart excision and anaesthetized by inhalation of 100% CO_2_. Deep anesthesia was confirmed by lack of response to otherwise painful stimuli. The mouse was then euthanised and the heart surgically removed via thoracotomy and placed in a Langendorff column. The isolated hearts were perfused sequentially at a constant flow rate of 3 ml/min with Ca^2+^-free solution containing (in mmol/l): 113 NaCl, 4.7 KCl, 1.2 MgSO_4_, 0.6 Na_2_HPO_4_, 0.6 KH_2_PO_4_, 12 NaHCO_3_, 10 KHCO_3_, 10 HEPES and 30 Taurine, pH 7.45 with NaOH and then an enzyme (collagenase type II; Worthington, Lakewood, NJ, USA) solution for 10 min. Perfusate temperature was maintained at 37 °C. After digestion, ventricles were cut into small pieces, and gently minced by gentle mechanical agitation with a Pasteur pipette. The isolated cardiomyocytes were suspended in 10 ml of stop buffer (Ca^2+^-free perfusion buffer with 5% bovine calf serum) and the Ca^2+^ concentration was increased gradually to 1.0 mM. Cardiomyocytes were kept in Tyrode’s solution containing (in mmol/l): 148 NaCl, 5.4 KCl, 1.0 MgCl_2_, 1.0 CaCl_2_, 0.4 NaH_2_PO_4_, 15 HEPES and 5.5 Glucose, pH 7.4. Cells were used within 8 h after isolation.

### Whole-cell patch clamp recordings

Macroscopic Ca^2+^ currents were recorded using the whole-cell patch-clamp configuration with the external recording solution of the following composition (in mmol/l): 140 TEA-Cl, 10 CsCl, 10 Glucose, 10 HEPES, 1.0 MgCl_2_, 1.2 CaCl_2_, 2.0 4-AP, pH 7.4 with CsOH. An internal pipette solution contained (in mmol/l): 20 TEA-Cl, 120 CsCl, 10 HEPES, 5 EGTA, 5 Mg-ATP, 0.3 GTP, pH 7.2 with CsOH. Patch pipettes had mean resistances of 1.5–3 MΩ. All recordings were performed at room temperature (22–24 °C). Assessment of *I*_Ca_ density was obtained by holding the cell at −80 mV, followed by stepping to voltages between −60 and +40 mV, in 10 mV steps, for 400 ms, with 5 s interpulse intervals.

NCX current was measured using an external solution containing (in mmol/l): 140 NaCl, 1.0 CaCl_2_, 1 MgCl_2_, 10 HEPES, 10 Glucose, 5 CsCl, 0.01 Nifedipine, 0.05 Oubain, 1 BaCl_2_, pH 7.4 (NaOH). The recording pipettes were filled with a solution containing (in mmol/l): 20 NaCl, 65 CsCl, 20 TEA-Cl, 6 CaCl_2_, 10 EGTA, 5 MgATP and 10 HEPES, pH 7.25 with CsOH. The peak NCX current density was measured at the 2000 ms test pulse to +60 mV from −80 mV holding potential and five sweeps before and after NiCl_2_ (5 mM) application were averaged and subtracted^52^.

Late Na^+^ current was measured using an external solution containing (in mmol/l): 135 NaCl, 5.4 KCl, 1.0 CaCl_2_, 1 MgCl_2_, 10 HEPES, 10 Glucose, 0.2 CdCl_2_, pH 7.4 (NaOH). The recording pipettes were filled with a solution containing (in mmol/l): 5 NaCl, 135 CsF, 10 EGTA, 5 MgATP and 5 HEPES, pH 7.2 with CsOH. The late tetrodotoxin-sensitive Na^+^ current density was measured at the end of a 500 ms test pulse to −30 mV from −90 mV holding potential and 20 sweeps before and after tetrodotoxin (20 μM) application were averaged and subtracted^41^. Currents were normalized to the cell capacitance and expressed in pA/pF.

### Action potential recording

Current clamp was used to measure adult cardiomyocyte action potential, the recording pipette solution contained (in mmol/l): 135 KCl, 1 MgCl_2_, 10 EGTA, 10 HEPES, and 5 Glucose, pH 7.2 with KOH. The bath solution contained (in mmol/l): 136 NaCl, 4 KCl, 1 CaCl_2_, 2 MgCl_2_, 0.2 CdCl_2_, 10 HEPES, 0.04 Tetrodotoxin and 10 Glucose, pH 7.4 with NaOH. Resting membrane potentials, action potential amplitudes, and action potential duration at 90% (APD90) repolarization were measured.

### Super-resolution scanning patch clamp

This method combines scanning ion-conductance microscopy (SICM) with cell-attached patch clamp technology for recording of ion channels at a particular subcellular location. A detailed description of this technique can be found in Bhargava et al.^25^ Briefly, after generating the topographical image of the cardiomyocyte surface with SICM, the pipette was moved to an area clear of cells or debris. At that coordinate, a custom-built program was used to clip the tip of the pipette against the bottom of the dish. The pipette resistance was continuously monitored and the clipping manoeuvre stopped once the current through the pipette reached the desired level. At that point, the pipette was repositioned to spatial coordinates that were selected based on the topography image recorded with the sharp pipette. The repositioned, clipped pipette was lowered at the chosen subcellular location (T-tubule or crest) to record Ca^2+^ channels in the cell-attached configuration. For recording of Ca^2+^ channels, cardiomyocytes were bathed in an external solution containing in (mmol/l): 120 K-gluconate, 25 KCl, 2 MgCl_2_, 1 CaCl_2_, 2 EGTA, 10 Glucose, 10 HEPES, pH 7.4 with NaOH. Pipettes were filled with an internal recording solution containing in (mmol/l): 90 BaCl_2_, 10 HEPES, 10 Sucrose, pH 7.4 with TEA-OH. Single LTCCs were identified and characterized by their voltage dependent properties. For this purpose, pulses from a holding potential of −80 mV were elicited to test potentials between −20 and +20 mV.

### Ca^2+^ imaging in isolated cardiomyocytes

Isolated ventricular myocytes were loaded for 12 min with x-Rhod-1/AM (Invitrogen Inc., Eugene, OR, USA) in Tyrode’s solution containing (in mmol/l): 140 NaCl, 4 KCl, 1.8 CaCl_2_, 1 MgCl_2_, 10 HEPES and 5.6 glucose followed by a 30 min wash in Tyrode’s solution. Fluorescent signals were acquired using a ×40 UVF objective (numerical aperture 1.0; Nikon, Tokyo, Japan), and single excitation wavelength microfluorimetry was performed using a PMT system (IonOptix Corp., Milton, MA, USA). Cells were paced at 1 Hz to achieve steady state, then paced either for 10 beats at 0.5 Hz and 1 Hz. Following background subtractions, data were calculated as the ratio of fluorescence intensity of x-Rhod-1 over baseline (*F*/*F*_0_). 10 mM Caffeine was used to determine the SR Ca^2+^ load after the cells were paced at 1 Hz and achieved steady state. To measure Ca spark, isolated ventricular myocytes were loaded with Fluo-8/AM and line scans were used to obtain Ca^2+^ spark data with confocal microscopy. Finally, ratiometric analysis to determine diastolic [Ca^2+^]_i_ was based on the method described by Li et al.^53^ Isolated ventricular myocytes were loaded with Fura-2/AM. The free calcium concentration were determined with the equation [Ca^2+^] = *K*_d_*β*(*R*−*R*_min_)/(*R*_max_−*R*), where *K*_d_ is the constant of 245 nM. *R* is the ratio of *F*_340_/*F*_380_. *R*_max_ and *R*_min_ values were determined after cells were treated with ionophore (4-Bromo A23187)/6 mM Ca^2+^ or ionophore/10 mM EGTA, respectively. *β* is the ratio of *F*_380_-EGTA/*F*_380_-Ca^2+^.

### Ca^2+^ leak in permeabilized cells

Ventricular myocytes were plated on glass-bottom dishes coated with 1 mg/ml laminin and maintained in bath solution containing (in mmol/l) 135 NaCl, 4 KCl, 1.8 CaCl_2_, 1 MgCl_2_, 10 HEPES, 1.2 NaH_2_PO_4_, and 10 glucose, pH 7.40, until used. Myocytes were permeabilized with 50 µg/ml saponin in 10 nM free [Ca^2+^] internal solution, containing (in mmol/l) 0.5 EGTA, 10 HEPES, 120 K-aspartate, 0.56 MgCl_2_, 5 Mg-ATP, 10 reduced glutathione, 5 phosphocreatine, 5 U/ml creatine phosphokinase, 8% dextran (Mr: 40,000), pH 7.2, and enough CaCl_2_ to adjust free [Ca^2+^] (MaxChelator). Ca^2+^ leak was recorded in permeabilized myocytes perfused with 70 nM free [Ca^2+^] internal solution supplemented with 0.025 mM Fluo-4 pentapotassium salt (Invitrogen), using a Zeiss LSM510 Meta confocal microscope with a ×40 1.2 NA water-immersion objective. Line-scan images were acquired at the sampling rate of 3 ms per line. After 30 s of basal recording, 10 mM caffeine was rapidly applied to measure SR load. Then, cells were perfused with 70 nM [Ca^2+^]_i_ internal solution again for 1 min, and perfused with 10 mM caffeine for the second SR Ca^2+^ load measurement.

### Excitation–contraction coupling gain

Whole-cell patch-clamp combined with Ca^2+^ transient recording experiments were performed to measure e–c coupling gain. Briefly, cells were bathed in a solution containing (in mM): 135 NaCl, 4 KCl, 1.8 CaCl_2_, 1 MgCl_2_, 10 HEPES, 1.2 NaH_2_PO_4_, and 10 glucose, 4 4-aminopyridine and 0.03 TTX, pH 7.4 with NaOH. The pipette internal solution contained (in mM): 100 Cs-Aspartate, 10 CsCl, 1 MgCl_2_, 5 MgATP, 0.5 Na_2_GTP, 10 HEPES, and 15 TEA·Cl, pH 7.2 with CsOH. Cells were voltage-clamped at −50 mV, depolarized from −40 mV to +60 mV at 10-mV increments for 300 ms, and repolarized to −50 mV between sweeps. Ca^2+^ transients triggered by Ca^2+^ currents were recorded at the same time. The Ca^2+^ indicator Fluo-4 pentapotassium salt (0.2 mM; Invitrogen) was added to the internal solution before the experiment.

### [^3^H]Ryanodine binding assays

Binding assays were carried out following a modified version of a protocol previously described^54, 55^. Whole heart homogenates were prepared as previously described^56^. In brief, frozen hearts were pulverized in liquid nitrogen and suspended in a buffer containing 0.9% NaCl, Tris-HCl 10 mM pH 6.8, 20 mM NaF, 2 µM leupeptin, 100 µM phenylmethylsulphonyl fluoride, 500 µM benzamidine, 100 nM aprotinin. This suspension was homogenized using a Teflon pestle and centrifuged at 1000×*g* for 10 min at 4 °C. Supernatants were aliquoted and stored at −80 °C until used. Protein concentrations were determined using the Bradford method (Bio-Rad). Binding mixtures were prepared containing 50 µg of protein from heart homogenates, 0.2 M KCl, 20 mM Na-HEPES pH 7.4, 6.5 nM [^3^H]Ryanodine (Perkin Elmer), 1 mM EGTA and enough CaCl_2_ to set free [Ca^2+^] between 100 nM to 100 μM. The Ca^2+^/EGTA ratio was calculated with MaxChelator (WEBMAXCLITE v1.15, http://maxchelator.stanford.edu). The binding reactions were incubated for 2 h at 37 °C, then filtered through Whatman GF/B filters and washed with distilled water in a Brandel M24-R Harvester. [^3^H]Ryanodine binding was determined by liquid scintillation and corrected for non-specific binding determined in the presence of 20 μM unlabeled ryanodine (MP Biomedicals). Maximum [^3^H]ryanodine binding corrected for RyR2 expression and EC_50_ were calculated with Hill’s equation using Origin 9 (Origin Lab).

### Mathematical simulations

Computational simulations were conducted in a model of the mouse ventricular myocyte using the formulation described by Morotti et al.^27^ To model the PKP2-KO mouse myocyte, the following changes were implemented in accordance with experimental observations: reduction of Calsequestrin in the SR to 45.3% of its concentration in the Control model; adjustment of the closing rate of the L-type Calcium channels to 75% of its original value and scaling down the current density of the L-type Calcium current to 50% of its original value, leading to a peak current 72% of the original (Control) model, the latter also in accordance with experimental findings; adjustment in the localization of L-type Calcium channels to model decreased expression in the T-tubules; RyR2 current adjustment to 60% of its original value to account for its observed reduced abundance. To assess e–c coupling gain, a holding potential of −50 mV was applied to the cell models for 600 s, to ensure steady state. Then, a test potential of −40 mV to +60 mV was applied for 150 ms. The peaks of the traces were identified and the e–c coupling gain was calculated as Gain = ([Ca^2+^]_i_/[Ca^2+^]_i,0_)/*I*_CaL,max_. In cases where Calcium discharges occurred spontaneously, the test potential was applied at least 500 ms after the previous discharge and at least 500 ms before the next discharge.

### Optical mapping

Mice were heparinized (heparin sodium, 0.5 U/g IP) and euthanized by CO_2_ inhalation followed by cervical dislocation. Hearts were quickly removed through a midline sternotomy and rinsed in a modified Tyrode’s solution containing (in mM): NaCl 130; NaHCO, 24; KH_2_PO_4_ 1.2; MgCl_2_ 1.0; glucose 11.1; KCl 4.7; and CaCl_2_ 1.8, equilibrated with a 95% O_2_–5% CO_2_ gas mixture. Hearts were rapidly cannulated and perfused with a constant pressure (50–60 mmHg) in a retrograde fashion via an aortic cannula with warm (37–39 °C) oxygenated modified Tyrode’s solution. Once connected to the Langendorff perfusion system, hearts were immersed in modified oxygenated Tyrode’s in a jacketed perfusion chamber where the temperature was controlled (37–39 °C) to ensure the absence of transmural temperature gradients^57, 58^. The excitation–contraction uncoupler Blebbistatin (Enzo Life Sciences,4 mg/l) was added to the perfusate to limit motion artifacts during optical recordings. Calcium-dependent fluorescent signals were recorded using a modified microscope (MVX10 Olympus) equipped for epifluorescent illumination. A 530 nm mounted LED (ThorLabs) was used for excitation, combined with a 593 ± 20 nm band pass emission filter. Images were acquired with a CMOS camera (SciMedia MiCAM ULTIMA) at 1000 frames per second with 14-bit resolution from a 100 × 100-pixel array. Hearts were loaded with the Ca^2+^ indicator Rhod-2 AM (Enzo Life Sciences, 40 mg per heart). Images were acquired from the RV free wall while pacing at 120 ms BCL. Baseline fluorescence images were acquired before dye loading and subtracted from the movies prior to data analysis. Activation maps for calcium transients were generated using custom software. Movies were signal averaged to improve signal-to-noise ratio and pixels with low signal-to-noise ratio were excluded from analysis. Duration of Ca^2+^ transients (CaD) was determined on a pixel by pixel basis from the time of 50% maximum fluorescence during the rising phase to the time point of 30, 50, and 70% recovery of Ca^2+^ to its original baseline. Calcium transient time-to-peak was quantified as the rise time from minimum to maximum transient amplitude.

### Super-resolution fluorescent microscopy

22-squared coverslips (Fisherbrand) were coated with 10 µg/ml laminin (BD Biosciences) for 30 min. Isolated adult cardiomyocytes were plated on the coverslips and allowed to attach to the surface for 45 min at 37 °C. Cells were fixed with 4% paraformaldehyde in PBS for 10 min and left in PBS until further processing for immunostaining. Samples were permeabilized with 0.1% Triton in PBS for 10 min at room temperature. Blocking was done with PBS containing 2% bovine serum albumin, 2% glycine, and 0.2% gelatin for 30 min. Primary antibodies rabbit Ca_V_1.2 (dilution 1:250, Alomone) and monoclonal mouse RyR2 (dilution 1:100, Thermofisher) were diluted in blocking solution and incubated for 1 h at room temperature. Primary antibodies were washed with PBS and secondary antibodies anti-mouse Alexa Fluor 647 (dilution 1:3000) and anti-rabbit Alexa Fluor 568 (dilution 1:5000, Life Technologies) were incubated for 30 min at room temperature. For SRFM imaging, coverslips were mounted on slides with imaging buffer: 200 mM mercaptoethylamine and an oxygen scavenging system: 0.4 mg/ml glucose oxidase, 0.02 mg/ml catalase and 10% (wt/wt) glucose. Samples were imaged in a custom-built microscope set up equipped with a Leica DM3000 microscope, a 556 nm and 640 laser (OEM Laser Systems) and an HCX PL APO ×100 NA = 1.47 OIL CORR TIRF objective. Total internal reflection fluorescence or highly inclined illumination modes were used to excite the samples and improve signal-to-noise ratio. A Dual-View (DV2-Photometrics) was used to image two colors simultaneously, side-by-side, onto a EM-CCD camera (Andor iXon+897). Two-color movies containing 2000 frames were processed using an ImageJ macro routine based on the QuickPALM plugin. The two-color image was split into its separate channels and each reconstructed at 20 nm using the QuickPALM parameters full-width half-maximum 4 and signal/noise ratio 4. Super-resolved clusters were defined as “lateral” when localized along the lateral membrane or “midsection” when localized intracellular away from the lateral membrane. For lateral clusters, a line (1 µm thick) was drawn along the membrane and a mask created in order to select only those clusters for further analysis. Midsection clusters were selected by creating a mask using the polygon section tool in ImageJ. Images were then processed with a smoothing filter, adjusted for brightness and contrast and filtered to a threshold to obtain a binary image. Cluster detection was performed using the function “Analyze particles” in ImageJ. Distances between clusters were obtained using the ImageJ function “Analyze particles” and a script written in Python that utilized the image processing packages “scikit-image” and “mahotas”.

### 2D transmission electron microscopy of the dyad

Mice were anesthetized with carbon dioxide inhalation, perfused with 2% paraformaldehyde and 2.5% glutaraldehyde in PBS and then euthanized by excision of the heart. The perfused heart was cut into 1 mm^3^ and placed in the same fixative solution at 4 °C overnight. Fixed mouse heart was processed with OTOTO method and embedded in Durcupan as described in our previous paper^23^. Briefly, after washing with 0.1 M PBS for 30 min at room temperature, the tissue was placed in 2% OsO_4_/1.5% potassium ferrocyanide in PBS for 1 h at room temperature, washed three times for 5 min in ddH_2_O at room temperature and then placed in a filtered solution of 1% thiocarbohydrazide (EMS) in ddH_2_O for 20 min at room temperature to allow for additional osmium staining. The tissue was then washed three times in ddH_2_O and then placed in 2% aqueous OsO_4_ for 30 min at room temperature. Finally, the tissue was washed three times in ddH_2_O and placed in 1% aqueous uranyl acetate at 4 °C overnight. The next day, tissue was washed three times in ddH_2_O. En bloc lead staining was performed to enhance membrane contrast^59^. A lead aspartate solution was made by dissolving 0.066 g of lead nitrate in 10 ml of 0.003 mg aspartic acid. The pH was adjusted to 5.5 with 1 N KOH, and the solution was placed in a 60 °C oven for 30 min. The lead aspartate solution was filtered, and the tissue was stained at 60 °C for 30 min. It was determined that this enhanced osmium staining protocol, combined with en bloc lead staining, was critical for enhancing membrane contrast. The sample was then washed three times in ddH_2_O, dehydrated in a series of ethanol solutions (30, 50, 70, 85, 95, 100, 100%; 10 min each, on ice) and then placed in ice-cold dry acetone for 10 min, followed by 10 min in acetone at room temperature. The sample was then gradually equilibrated with Durcupan ACM Araldite embedding resin (Electron Microscopy Sciences, EMS, PA) by placing in 25% Durcupan/acetone for 2 h, 50% Durcupan/acetone 2 h, 75% Durcupan/acetone for 2 h, and 100% Durcupan overnight. The tissue was then embedded in fresh 100% Durcupan and placed in a 60 °C oven for 48 h to allow Durcupan polymerization and complete the embedding procedure). Sections 60 nm thin were cut and mounted on 200 mesh copper grids (Electron Microscopy Sciences, Hatfield, PA). Images were acquired at ×8800 and ×66,000 magnification using a Philips CM-12 electron microscope (FEI; Eindhoven, The Netherlands) equipped with a Gatan (4k × 2.7k) digital camera (Gatan, Inc., Pleasanton, CA). Upon selection of only dyads presenting clear membrane contours, a dataset composed of 30 (control), 41 (PKP2-cKO 1) and 41 (PKP2-cKO 2) dyads imaged at ×66,000 was processed and analyzed in the following way: contours of the T-tubule and the adjacent sarcoplasmic reticulum were traced manually, thus generating a 2D binary mask for each dyad, where pixels corresponding to the membranes were assigned a value of 1 (white) and the rest of the image received a value of zero (black). Sarcoplasmic reticulum membrane was defined as boundary 1 (in red) and T-tubule membrane was defined as boundary 2 (in blue) using the Matlab function “bwboundaries.” Then, for each point in the boundary 2, we identified the shortest distance to the opposing one. Mean and minimum values of these distances were obtained and used for comparison.

### Serial block face scanning electron microscopy (SBF-SEM)

The sample block was trimmed and thin sections were cut on slot grids to identify the area of interest. The sample block was then mounted on the SEM sample holder using double sided carbon tape (EMS). Colloidal silver paint (EMS) was used to electrically ground the exposed edges of the tissue block. The entire surface of the specimen was sputter coated with a thin layer of gold/palladium and the tissue was imaged using a FEI Teneo VolumeScope which is a scanning electron microscope equipped with a microtome unit. The system was set to cut sections with 20 nm thickness, imaged in low vacuum mode and images were recorded after each round of section from the block face using the SEM beam at 3.09 keV, 50 pA and pixel dwell time of 4 μs. Each raw image had the following dimensions: *X*: 7 nm per pixel; *Y*: 7 nm per pixel and *Z*: 20 nm per slice. Data acquisition occurred in an automated way using MAPs software. A volume of roughly 15 × 12 × 0.8 µm^3^ dimensions was obtained from the tissue block. Contours of the present T-tubule network and the lateral membrane of a cardiac myocyte were manually traced on all the virtual slices and their 3D rendered models obtained. The manually traced 3D mask of the T-tubule network was projected along the *Z*-axis of the image stack, thresholded and skeletonized (Matlab function ‘bwmorph’) as in our previous study^24^. The spatial orientation of the skeletons was analyzed using the ‘directionality’ plug-in in ImageJ as in Wagner et al.^60^ Network components were color-coded according to the local angle of the skeleton relative to the long axis of the cardiomyocyte (0°) and a histogram of orientations was obtained. Segmentation and visualization were performed in Amira (FEI). ImageJ and Matlab (Mathworks, Natick, MS, USA) equipped with the Image Processing toolbox were used for quantitative analysis.

### Statistics

Data are represented as mean ± SEM. Significance was calculated using two-sided Student’s *t*-test for two group comparisons or one-way ANOVA with Bonferroni post test for more than two different groups, as indicated in the figure legends. Analysis was done using the GraphPad Prism 6 and SPSS 23.0, IBM packages. All statistical comparisons involved groups of at least six samples and, in the case of cells in isolation (patch clamp), cells were obtained from at least two animals. Specifics are provided in the figure legends. Sample sizes were determined by animal availability, cost and degree of difficulty in data generation, and previous experience by us and other laboratories in standards of practice. The assumption was made that variations within the group would follow a normal distribution and as such, unless specifically noted in the figure legend, parmetric statistics were used. Variance was similar between groups being statistically compared. Criteria for inclusion (or exclusion) were only based on genotype and age of the animals (both genders were included). Given that two specific genotypes were compared, the animals needed to be genotyped prior to entering the experimental arms, and that information was acquired by the experimental operators (as well as the day of injection), there was no blinding and/or randomization of the individual animals; operators knew the genotype and the number of days after tamoxifen injection for the sample or animal under study.

### Data availability

All relevant data are available from the authors. RNAseq data have been deposited in Figshare.com under accession code 5034932.v1 (https://doi.org/10.6084/m9.figshare.5034932.v1). Primers used for RT-PCR have been deposited in Figshare.com under accession code 5034923.v1 (https://doi.org/10.6084/m9.figshare.5034923.v1).

## Electronic supplementary material

Supplementary Information

Supplementary movie 1

## References

[1] Corrado D, Link MS, Calkins H (2017). Arrhythmogenic right ventricular cardiomyopathy. N. Engl. J. Med..

[2] Philips B, Cheng A (2016). 2015 update on the diagnosis and management of arrhythmogenic right ventricular cardiomyopathy. Curr. Opin. Cardiol..

[3] Groeneweg JA (2015). Clinical presentation, long-term follow-up, and outcomes of 1001 arrhythmogenic right ventricular dysplasia/cardiomyopathy patients and family members. Circ. Cardiovasc. Genet..

[4] Basso C, Corrado D, Marcus FI, Nava A, Thiene G (2009). Arrhythmogenic right ventricular cardiomyopathy. Lancet.

[5] Delmar M, McKenna WJ (2010). The cardiac desmosome and arrhythmogenic cardiomyopathies: from gene to disease. Circ. Res..

[6] Lombardi R (2016). Cardiac fibro-adipocyte progenitors express desmosome proteins and preferentially differentiate to adipocytes upon deletion of the desmoplakin gene. Circ. Res..

[7] Chen SN (2014). The hippo pathway is activated and is a causal mechanism for adipogenesis in arrhythmogenic cardiomyopathy. Circ. Res..

[8] Lombardi R (2011). Nuclear plakoglobin is essential for differentiation of cardiac progenitor cells to adipocytes in arrhythmogenic right ventricular cardiomyopathy. Circ. Res..

[9] Lombardi R (2009). Genetic fate mapping identifies second heart field progenitor cells as a source of adipocytes in arrhythmogenic right ventricular cardiomyopathy. Circ. Res..

[10] Lombardi R, Marian AJ (2010). Arrhythmogenic right ventricular cardiomyopathy is a disease of cardiac stem cells. Curr. Opin. Cardiol..

[11] Agullo-Pascual E, Cerrone M, Delmar M (2014). Arrhythmogenic cardiomyopathy and Brugada syndrome: diseases of the connexome. FEBS Lett..

[12] Dubash AD (2016). Plakophilin-2 loss promotes TGF-beta1/p38 MAPK-dependent fibrotic gene expression in cardiomyocytes. J. Cell Biol..

[13] Agullo-Pascual E (2014). Super-resolution imaging reveals that loss of the C-terminus of connexin43 limits microtubule plus-end capture and NaV1.5 localization at the intercalated disc. Cardiovasc. Res..

[14] Faggioni M (2013). Accelerated sinus rhythm prevents catecholaminergic polymorphic ventricular tachycardia in mice and in patients. Circ. Res..

[15] Makara MA (2014). Ankyrin-g coordinates intercalated disc signaling platform to regulate cardiac excitability *in vivo*. Circ. Res..

[16] Garcia-Gras E (2006). Suppression of canonical Wnt/beta-catenin signaling by nuclear plakoglobin recapitulates phenotype of arrhythmogenic right ventricular cardiomyopathy. J. Clin. Invest..

[17] Ter Keurs HE, Boyden PA (2007). Calcium and arrhythmogenesis. Physiol. Rev..

[18] Chopra N (2009). Ablation of triadin causes loss of cardiac Ca2+ release units, impaired excitation-contraction coupling, and cardiac arrhythmias. Proc. Natl. Acad. Sci. USA.

[19] Chopra N, Knollmann BC (2013). Triadin regulates cardiac muscle couplon structure and microdomain Ca(2+) signalling: a path towards ventricular arrhythmias. Cardiovasc. Res..

[20] Camors E, Mohler PJ, Bers DM, Despa S (2012). Ankyrin-B reduction enhances Ca spark-mediated SR Ca release promoting cardiac myocyte arrhythmic activity. J. Mol. Cell. Cardiol..

[21] Kline CF, Scott J, Curran J, Hund TJ, Mohler PJ (2014). Ankyrin-B regulates Cav2.1 and Cav2.2 channel expression and targeting. J. Biol. Chem..

[22] Agullo-Pascual E (2013). Super-resolution fluorescence microscopy of the cardiac connexome reveals plakophilin-2 inside the connexin43 plaque. Cardiovasc. Res..

[23] Leo-Macias A (2016). Nanoscale visualization of functional adhesion/excitability nodes at the intercalated disc. Nat. Commun..

[24] Leo-Macias A, Liang FX, Delmar M (2015). Ultrastructure of the intercellular space in adult murine ventricle revealed by quantitative tomographic electron microscopy. Cardiovasc. Res..

[25] Bhargava A (2013). Super-resolution scanning patch clamp reveals clustering of functional ion channels in adult ventricular myocyte. Circ. Res..

[26] Sanchez-Alonso JL (2016). Microdomain-specific modulation of L-type calcium channels leads to triggered ventricular arrhythmia in heart failure. Circ. Res..

[27] Morotti S, Edwards AG, McCulloch AD, Bers DM, Grandi E (2014). A novel computational model of mouse myocyte electrophysiology to assess the synergy between Na+ loading and CaMKII. J. Physiol..

[28] Watanabe H (2009). Flecainide prevents catecholaminergic polymorphic ventricular tachycardia in mice and humans. Nat. Med..

[29] Hwang HS (2011). Inhibition of cardiac Ca2+ release channels (RyR2) determines efficacy of class I antiarrhythmic drugs in catecholaminergic polymorphic ventricular tachycardia. Circ. Arrhythm. Electrophysiol..

[30] Cerrone M (2012). Sodium current deficit and arrhythmogenesis in a murine model of plakophilin-2 haploinsufficiency. Cardiovasc. Res..

[31] Marcus FI (2010). Diagnosis of arrhythmogenic right ventricular cardiomyopathy/dysplasia: proposed modification of the task force criteria. Circulation.

[32] Rizzo S (2012). Intercalated disc abnormalities, reduced Na+ current density and conduction slowing in desmoglein-2 mutant mice prior to cardiomyopathic changes. Cardiovasc. Res..

[33] Kim C (2013). Studying arrhythmogenic right ventricular dysplasia with patient-specific iPSCs. Nature.

[34] Nadadur RD (2016). Pitx2 modulates a Tbx5-dependent gene regulatory network to maintain atrial rhythm. Sci. Transl. Med..

[35] Shekhar A (2016). Transcription factor ETV1 is essential for rapid conduction in the heart. J. Clin. Invest..

[36] Chen H (2013). Mechanism of calsequestrin regulation of single cardiac ryanodine receptor in normal and pathological conditions. J. Gen. Physiol..

[37] Terentyev D (2008). Modulation of SR Ca release by luminal Ca and calsequestrin in cardiac myocytes: effects of CASQ2 mutations linked to sudden cardiac death. Biophys. J..

[38] Denis A (2014). Diagnostic value of isoproterenol testing in arrhythmogenic right ventricular cardiomyopathy. Circ. Arrhythm. Electrophysiol..

[39] Ermakov S, Gerstenfeld EP, Svetlichnaya Y, Scheinman MM (2017). Use of flecainide in combination antiarrhythmic therapy in patients with arrhythmogenic right ventricular cardiomyopathy. Heart Rhythm.

[40] Cerrone M (2014). Missense mutations in plakophilin-2 cause sodium current deficit and associate with a brugada syndrome phenotype. Circulation.

[41] Lubkemeier I (2013). Deletion of the last five C-terminal amino acid residues of connexin43 leads to lethal ventricular arrhythmias in mice without affecting coupling via gap junction channels. Basic Res. Cardiol..

[42] Danik S (2002). Correlation of repolarization of ventricular monophasic action potential with ECG in the murine heart. Am. J. Physiol. Heart Circ. Physiol..

[43] Mitchell GF, Jeron A, Koren G (1998). Measurement of heart rate and Q-T interval in the conscious mouse. Am. J. Physiol..

[44] Ram R, Mickelsen DM, Theodoropoulos C, Blaxall BC (2011). New approaches in small animal echocardiography: imaging the sounds of silence. Am. J. Physiol. Heart Circ. Physiol..

[45] Brittain E, Penner NL, West J, Hemnes A (2013). Echocardiographic assessment of the right heart in mice. J. Vis. Exp.

[46] Dobin A (2013). STAR: ultrafast universal RNA-seq aligner. Bioinformatics.

[47] Anders S, Pyl PT, Huber W (2015). HTSeq–a Python framework to work with high-throughput sequencing data. Bioinformatics.

[48] Ringner M (2008). What is principal component analysis?. Nat. Biotechnol..

[49] Huang DW (2007). DAVID bioinformatics resources: expanded annotation database and novel algorithms to better extract biology from large gene lists. Nucleic Acids Res..

[50] Huang da W, Sherman BT, Lempicki RA (2009). Systematic and integrative analysis of large gene lists using DAVID bioinformatics resources. Nat. Protoc..

[51] Lin X (2011). Subcellular heterogeneity of sodium current properties in adult cardiac ventricular myocytes. Heart Rhythm.

[52] Beuckelmann DJ, Wier WG (1989). Sodium-calcium exchange in guinea-pig cardiac cells: exchange current and changes in intracellular Ca2+. J. Physiol..

[53] Li Q, Altschuld RA, Stokes BT (1987). Quantitation of intracellular free calcium in single adult cardiomyocytes by fura-2 fluorescence microscopy: calibration of fura-2 ratios. Biochem. Biophys. Res. Commun..

[54] Helms AS (2016). Genotype-dependent and independent calcium signaling dysregulation in human hypertrophic cardiomyopathy. Circulation.

[55] Loaiza R (2013). Heterogeneity of ryanodine receptor dysfunction in a mouse model of catecholaminergic polymorphic ventricular tachycardia. Circ. Res..

[56] Benkusky NA (2007). Intact beta-adrenergic response and unmodified progression toward heart failure in mice with genetic ablation of a major protein kinase A phosphorylation site in the cardiac ryanodine receptor. Circ. Res..

[57] Gutstein DE (2001). Conduction slowing and sudden arrhythmic death in mice with cardiac-restricted inactivation of connexin43. Circ. Res..

[58] Morley GE (2005). Reduced intercellular coupling leads to paradoxical propagation across the Purkinje-ventricular junction and aberrant myocardial activation. Proc. Natl. Acad. Sci. USA.

[59] Walton J (1979). Lead asparate, an en bloc contrast stain particularly useful for ultrastructural enzymology. J. Histochem. Cytochem..

[60] Wagner E (2012). Stimulated emission depletion live-cell super-resolution imaging shows proliferative remodeling of T-tubule membrane structures after myocardial infarction. Circ. Res..

