# Supplementary material for: Plakophilin-2 is required for transcription of genes that control calcium cycling and cardiac rhythm
Source: Nat Commun. 2017 Jul 24;8:106. doi: 10.1038/s41467-017-00127-0 (PMC5524637; doi:10.1038/s41467-017-00127-0)
Supplement: Supplementary file 1 — Supplementary Information [file 41467_2017_127_MOESM1_ESM.pdf]

File Name: Supplementary Information
Description: Supplementary Figures and Supplementary Tables.

File Name: Supplementary Movie 1
Description: Serial Block-Face Scanning Electron Microscopy analysis of adult ventricular tissue obtained from a PKP2-cKO mouse 21 dpi. Sergmentation analysis highlights the spatial orientation of the T-tubular network. Object volume: 15x12x0.8 μm^3. See also Figure 5 in the manuscript.

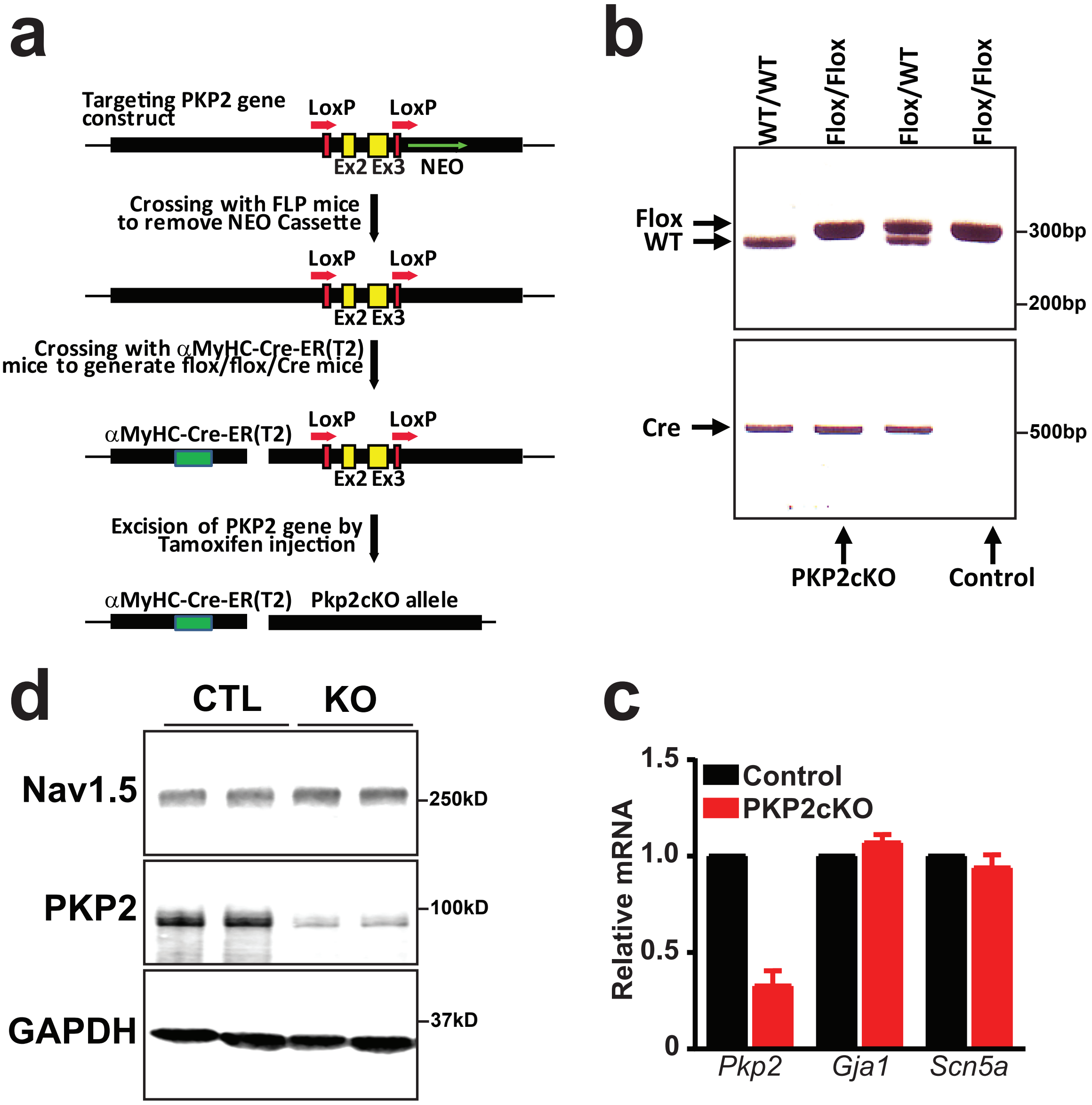

Supplementary Figure 1: Generation of PKP2-cKO mice. (**a**) Two forward loxP sites were designed and introduced into the construct flanking mouse PKP2 exons 2 and 3, with a downstream neomycin selection cassette. The linearized targeting construct was electroporated into C57B/6 derived ES cells and the resultant ES cell clones were identified. The confirmed positive ES cells were injected into isogenic blastocyts, and microinjected into the foster mice. The neo cassette was excised by crossing the F1 heterozygous mice with FRT mice. The PKP2 flox/flox mice were mated to αMyHC-Cre-ER(T2) mice to obtain flox/flox/Cre+ mice which contains α myosin heavy chain promoter and the ligand binding domain of the human estrogen receptor. Binding of Tamoxifen to the ER induces the cardiomyocyte specific Cre-mediated deletion of PKP2 gene. (**b**) The genotype of PKP2-cKO mice was identified by PCR screening. The flox/flox/cre+ and flox/flox/cre- mice were selected for the experiments. (**c**) Relative expression of PKP2, Gja1 and Scn5a mRNAs in controls and PKP2-cKO mice (n=3 in both groups). (**d**) Western blots showing expression of PKP2 in control and drastically reduced expression in PKP2-cKO hearts 2 weeks after the first tamoxifen injection. GAPDH was used as loading control.

Supplementary Fig. 1

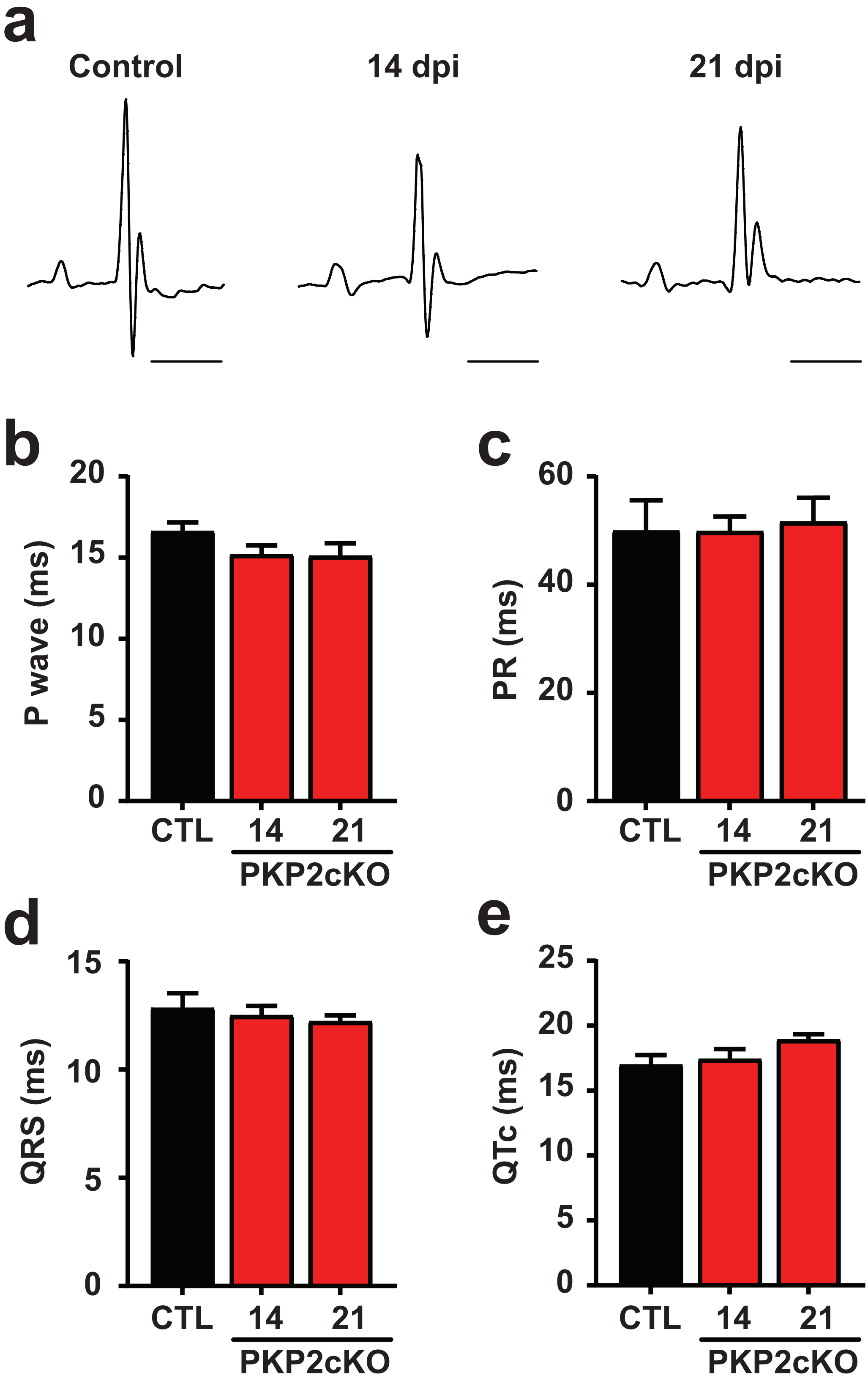

Supplementary Figure 2: ECG characteristics of PKP2-cKO mice. (**a**) Representative examples of Lead II ECG traces recorded in control (n=6), PKP2-cKO at 14 dpi (n=7), and at 21dpi (n=7) anesthetized mice. Scale = 50 ms. (**b-e**). Bar graphs depicting PR interval, P wave, QRS interval and QTc durations in controls (CTL, black, left) and PKP2-cKO mice (red; 14 dpi and 21 dpi). No statistical differences were observed among groups

Supplementary Fig. 2

a

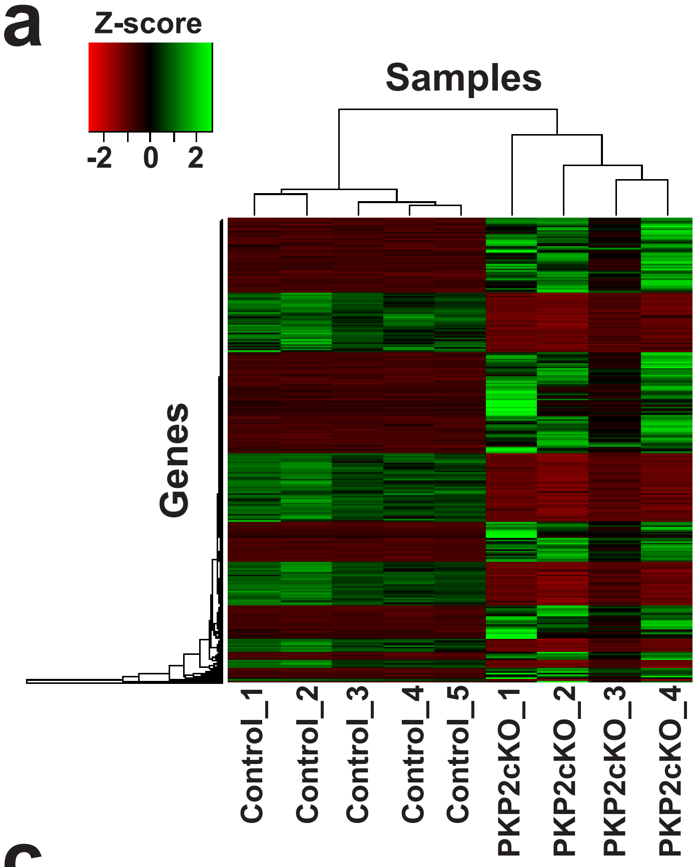

b

KEGG pathways	Genes	Log_2FC	FDR
**Insulin signaling pathway**	Crkl	-1.7	1.0×10^{-15}
	Sorbs1	-3.4	1.0×10^{-15}
	Tsc1	-1.5	1.0×10^{-15}
	Sos2	-1.7	1.0×10^{-15}
	Cbl	-2.5	2.7×10^{-14}
	Prkaa2	-1.5	2.3×10^{-14}
	Ppp1r3a	-2.3	1.4×10^{-12}
	Ppp1cb	-1.7	5.8×10^{-8}
	Ppargc1a	-1.9	1.6×10^{-14}
	Irs1	-2.8	1.0×10^{-15}
**Adipocytokine signaling pathway**	Ppara	-3.6	1.0×10^{-15}
	Cpt1b	-1.9	4.7×10^{-15}
	Prkaa2	-1.6	1.0×10^{-15}
	Ppargc1a	-1.9	1.6×10^{-14}
	Irs1	-2.8	1.0×10^{-15}
	Acsl6	-1.6	8.4×10^{-12}
**GnRH signaling pathway**	Adcy1	-1.8	8.5×10^{-7}
	Plcb4	-1.5	1.0×10^{-15}
	Adcy9	-3.5	1.0×10^{-15}
	Map3k3	-1.7	1.0×10^{-15}
	Sos2	-1.7	1.0×10^{-15}
	Cacna1c	-1.7	5.7×10^{-10}
	Cacna1s	-2.2	1.0×10^{-15}

c

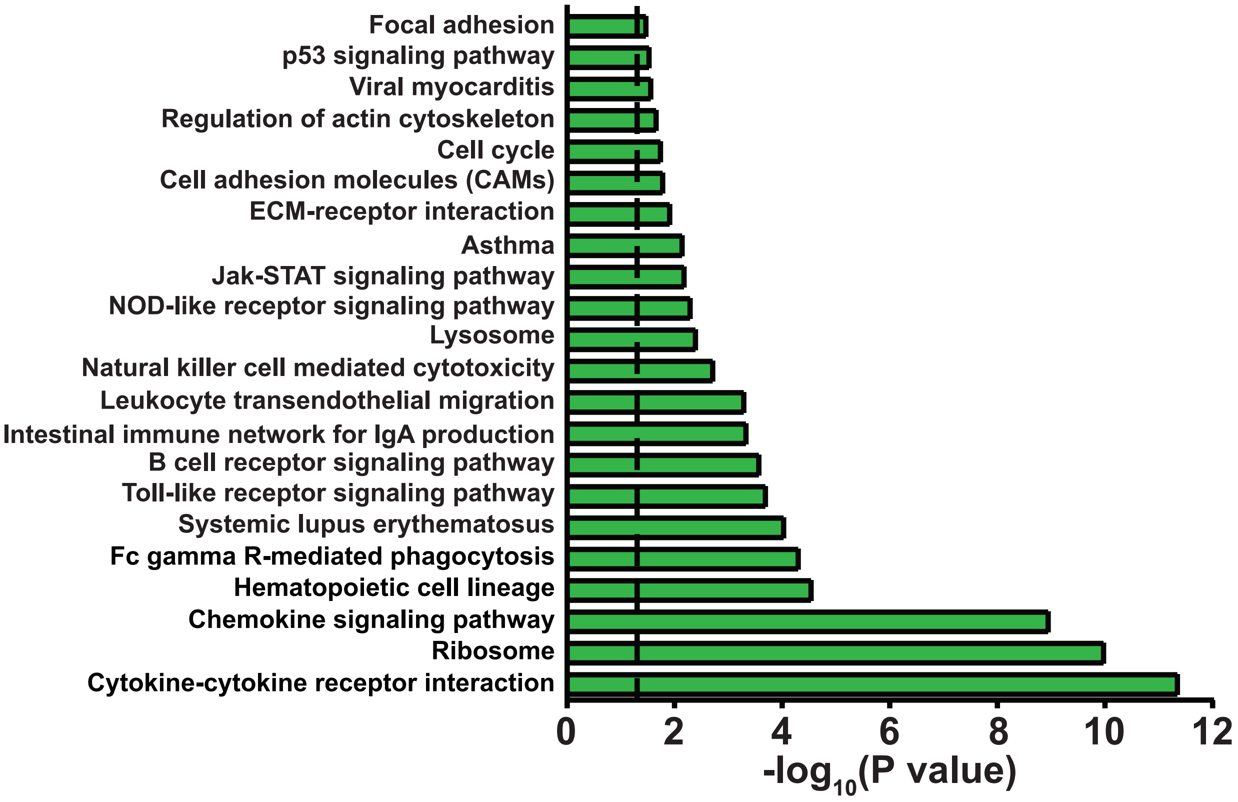

Supplementary Figure 3: RNASeq data. (**a**) Hierarchical clustering heatmap of the 1215 genes with the largest significance variance from 5 controls (CTRL) and 4 PKP2-cKO samples at 21 dpi shows good clustering of samples by genotype. (**b**) Specific genes, and scores, in gene networks that were identified by KEGG analysis to be downregulated with high statistical significance, and that are relevant to intracellular and paracrine signaling as well as cell metabolism. (**c**) KEGG analysis of upregulated transcripts in PKP2-cKO hearts 21 dpi, showing specific functional networks.

Supplementtray Fig. 3

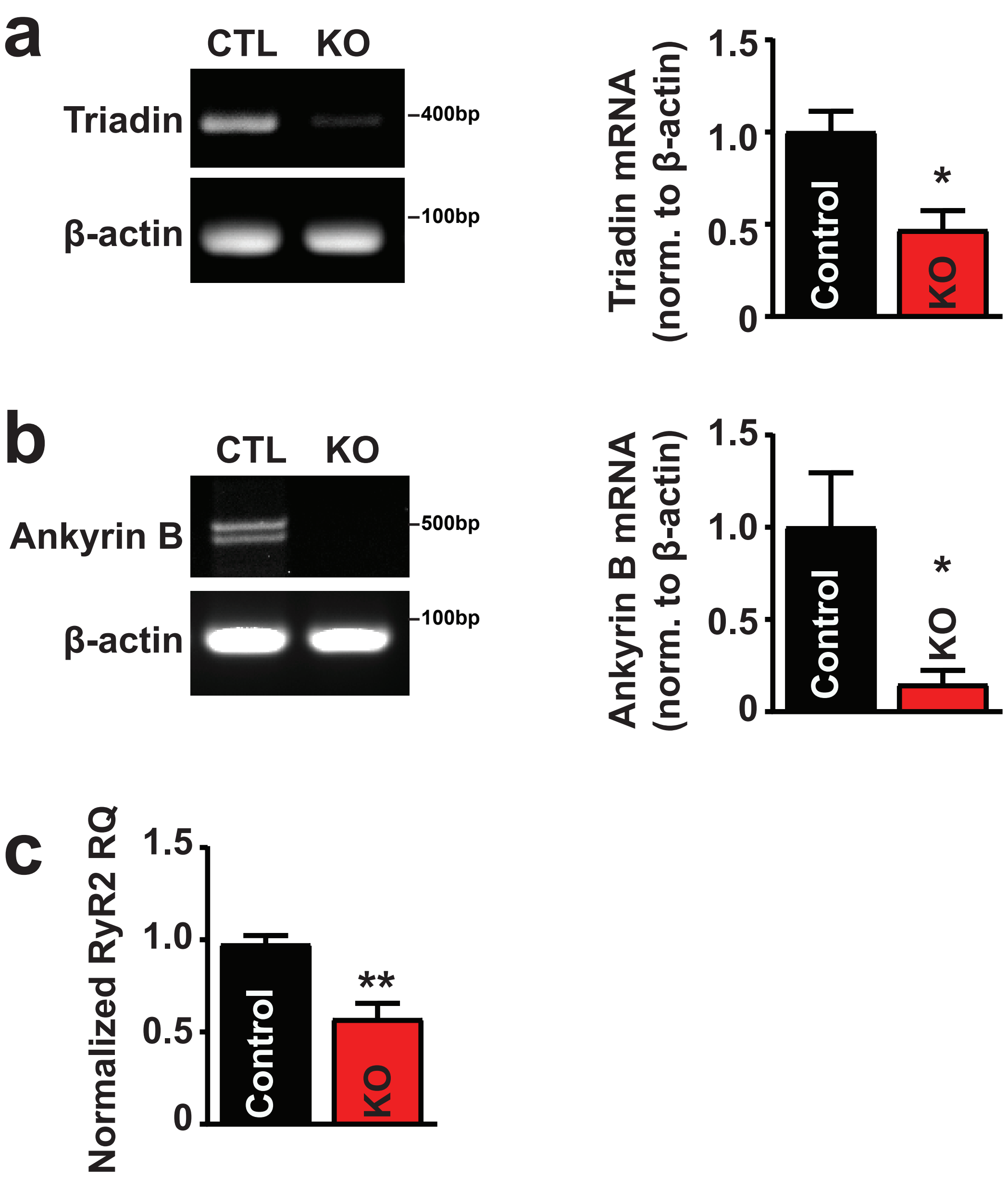

Supplementary Figure 4: Down-regulation of transcripts involved in calcium signaling. RT-PCR showing downregulation of transcripts of Triadin (**a**), and Ankyrin-B (**b**) in PKP2-CKO ventricular cardiomyocytes at 21 dpi. (c) Abundance of RyR2 transcript was estimated by qt-RT-PCR. Normalized expression relative quantification (RQ) was calculated as RQ= $2^{-\Delta\Delta CT}$

Supplementray Fig. 4

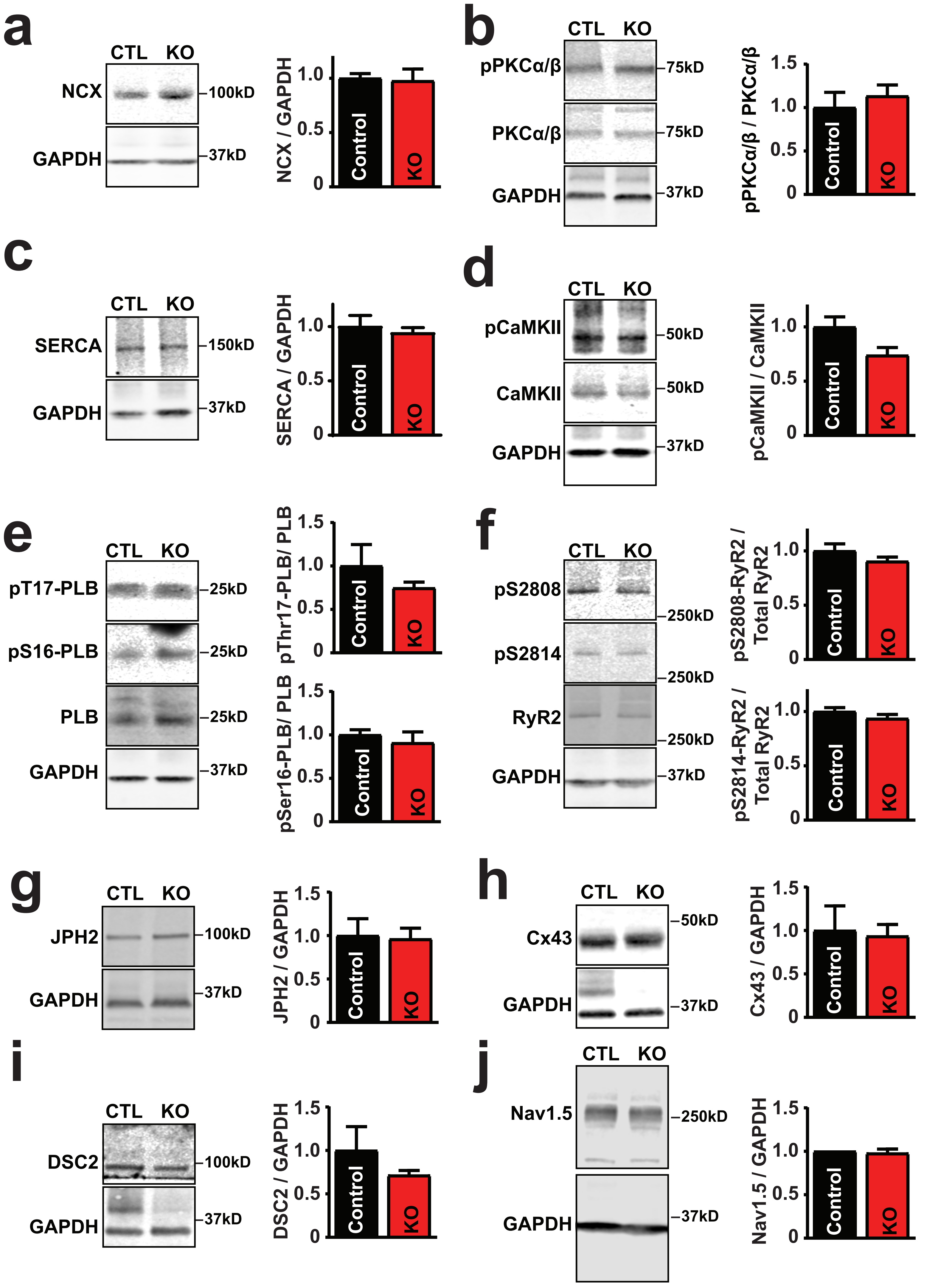

Supplementary Fig. 5

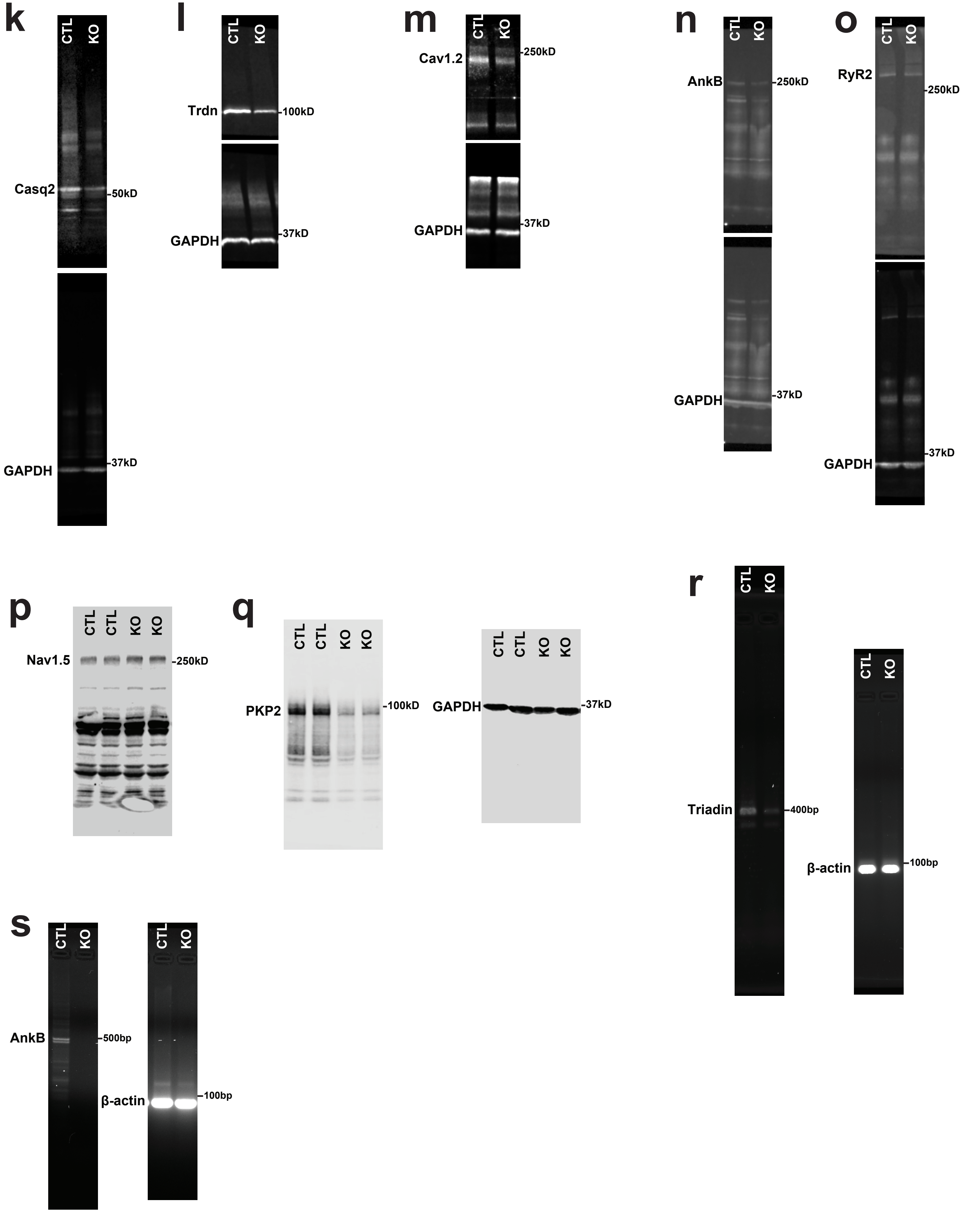

Supplementary Fig. 5

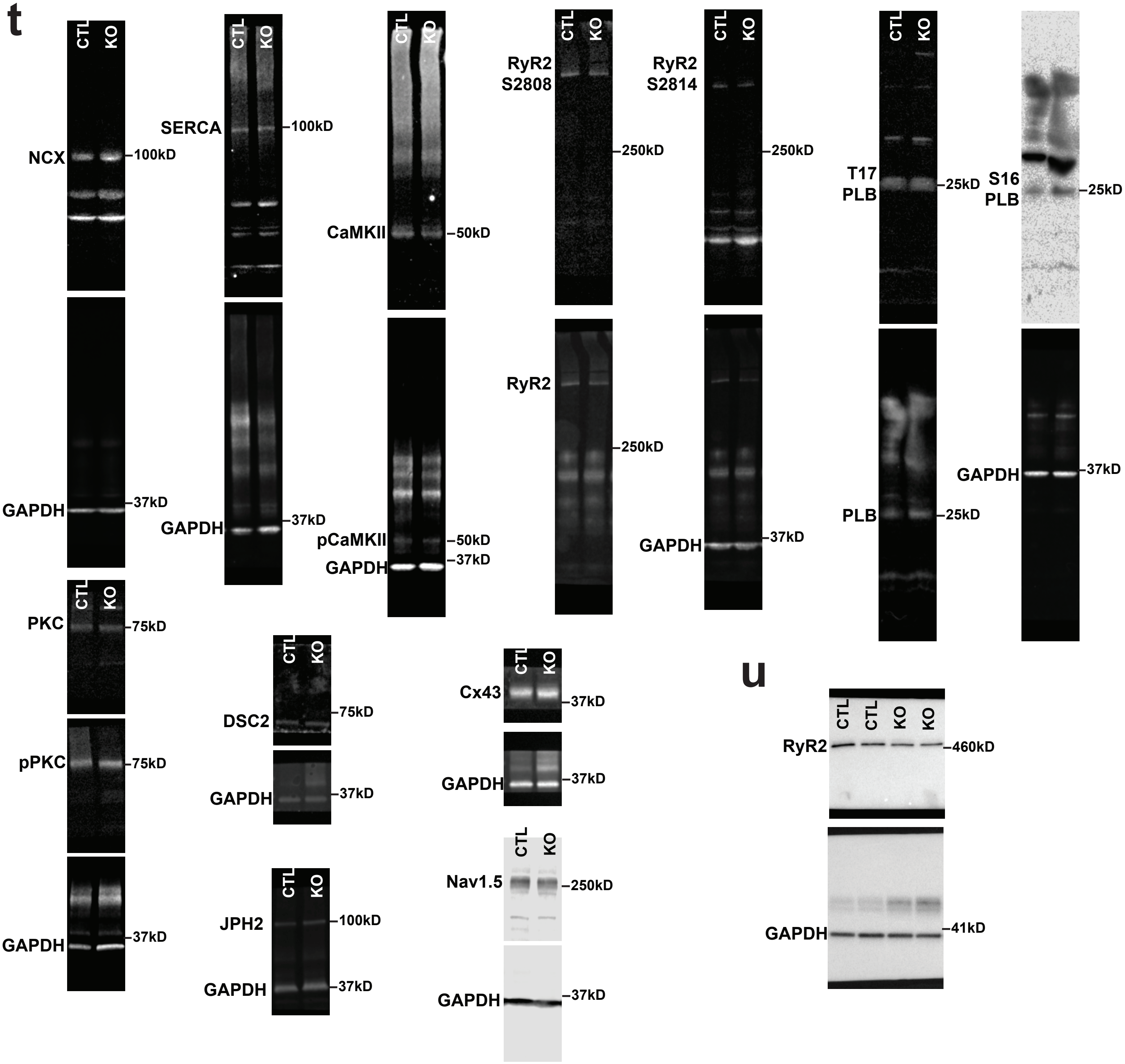

Supplementary Figure 5: Expression of proteins involved in calcium signaling in PKP2-cKO hearts (21 dpi). Western blots for of **a**) NCX, **b**) PKC (including phospho-form), **c**) SERCA, **d**) CaMKII (including phospho-form), **e**) PLB (including phospho-forms), **f**) RyR2 (including phospho-forms), **g**) junctophilin-2 (JPH2), **h**) Connexin43 (Cx43), **i**) desmocollin-2 (DSC2), and **j**) NaV1.5 in control (CTL) and PKP2-cKO (KO) ventricular lysates (n=6 for each condition). Representative examples are on the left and bar graph with combined results on the right. Full lanes of Western blots shown in Figure 4 of the main manuscript are presented in panels **k** (Casq2), **l** (Trdn), **m** (CaV1.2), **n** (AnkB) and **o** (RyR2). Panels **p** (Nav1.5) and **q** (Pkp2), full lane representation of blots shown in Supplementary Figure 1d. panels **r** (Trdn) and **s** (AnkB), full lane representation of gels shown in Supplementary Figure 4a-b. Panel **t,** full lane representation of blots shown in Supplementary Figure 5a-j. Panel **u,** full lane representation of blots shown in Supplementary Figure 7a.

Supplementary Fig. 5

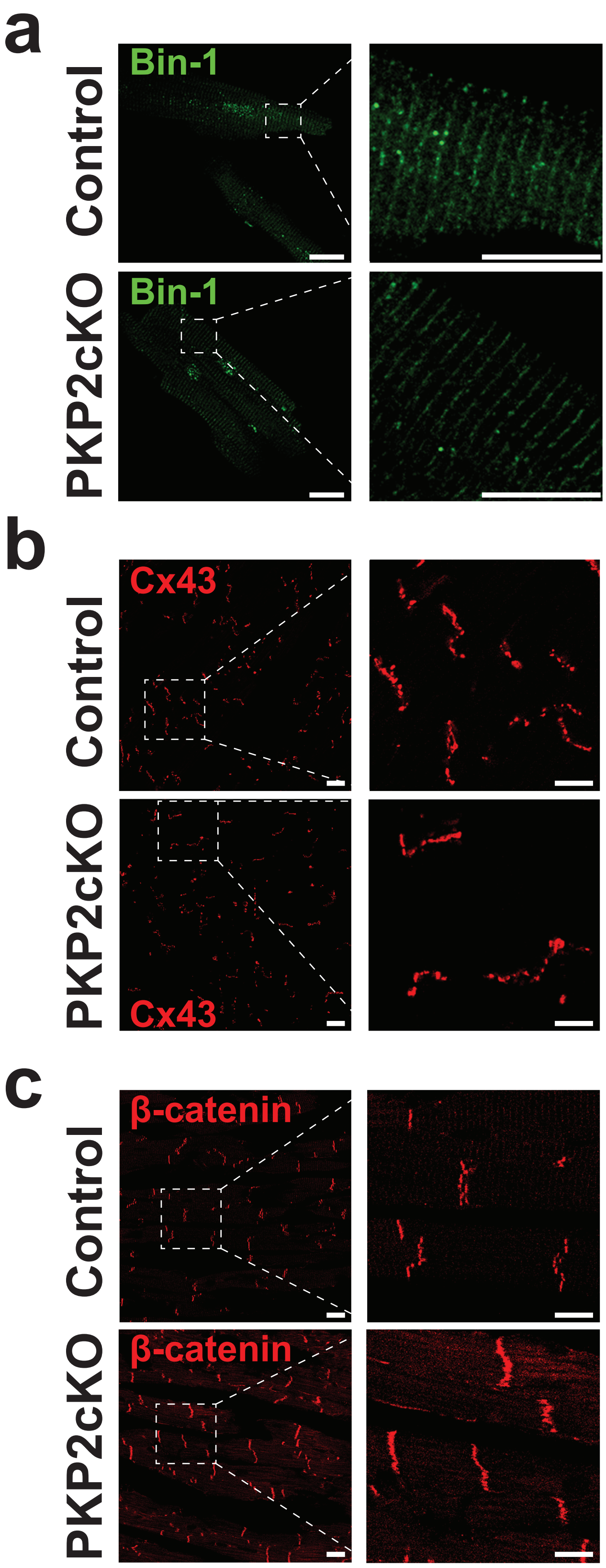

Supplementary Figure 6: Immunolocalization of Bin-1, Cx43 and β-catenin in PKP2-cKO cardiomyocytes. Immunostaining of Bin-1 (**a**), Cx43 (**b**) and β-catenin (**c**) showing absence of differences in control and PKP2-cKO cardiomyocytes. Scale bar = 20 μm. Magnificient images scale bar = 10 μm.

Supplementary Fig. 6

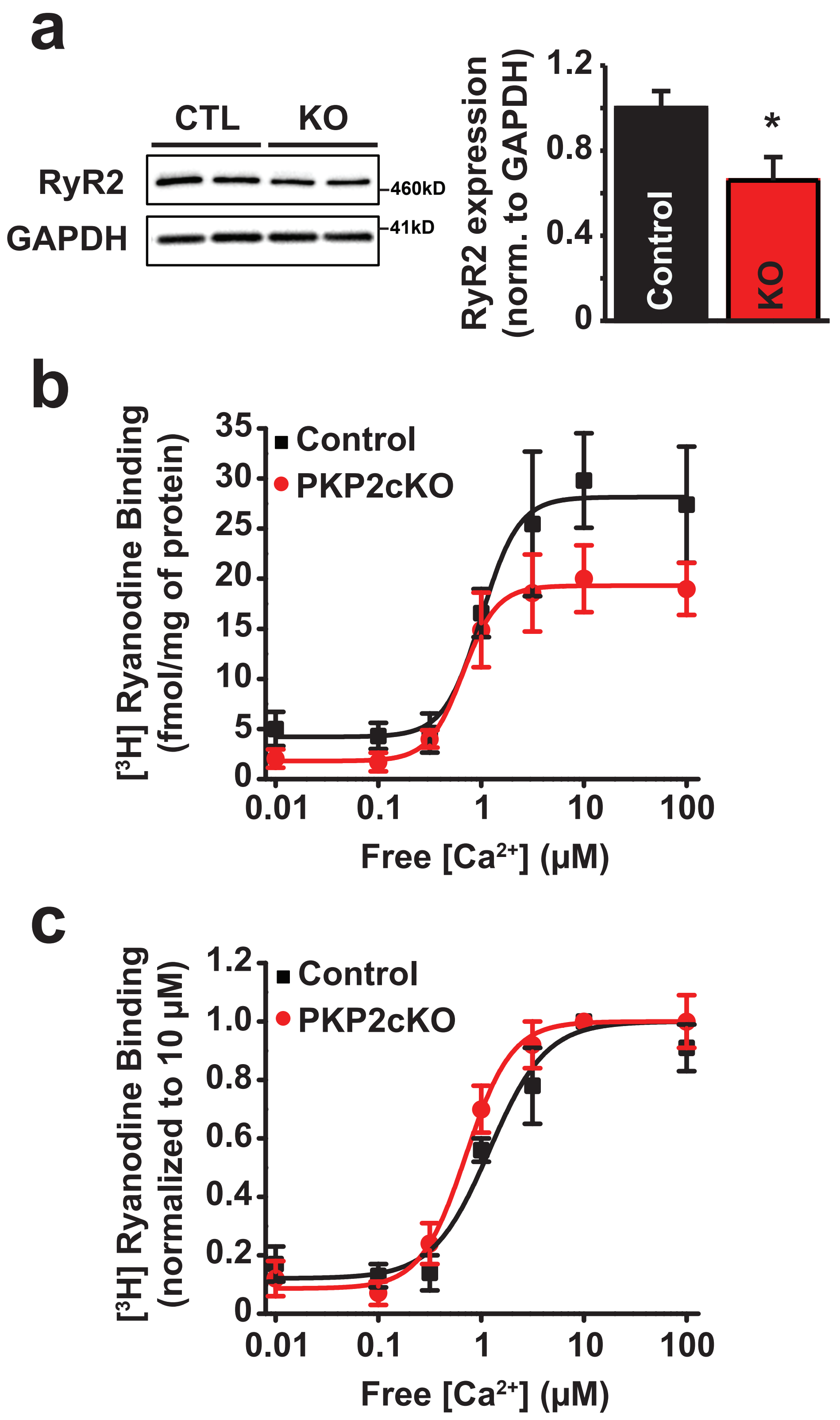

Supplementary Figure 7: Ryanodine receptor binding assay. (**a**) Expression of RyR2 confirmed by western blot in the same samples used for the binding assays. t-test, * $p<0.05$ versus control. (**b**) Ryanodine binding curves to control (black line and squares; n=5) and PKP2-cKO at 21dpi (red line and circles; n=6) of RyR2 shows a lower Bmax in PKP2-cKO samples. (**c**) Hill fitting of the Ryanodine binding assay in control (n=5) and PKP2-cKO at 21dpi (n=6) shows a modest and non-significant shift of the calcium sensitivity for RyR2 in PKP2-cKO hearts.

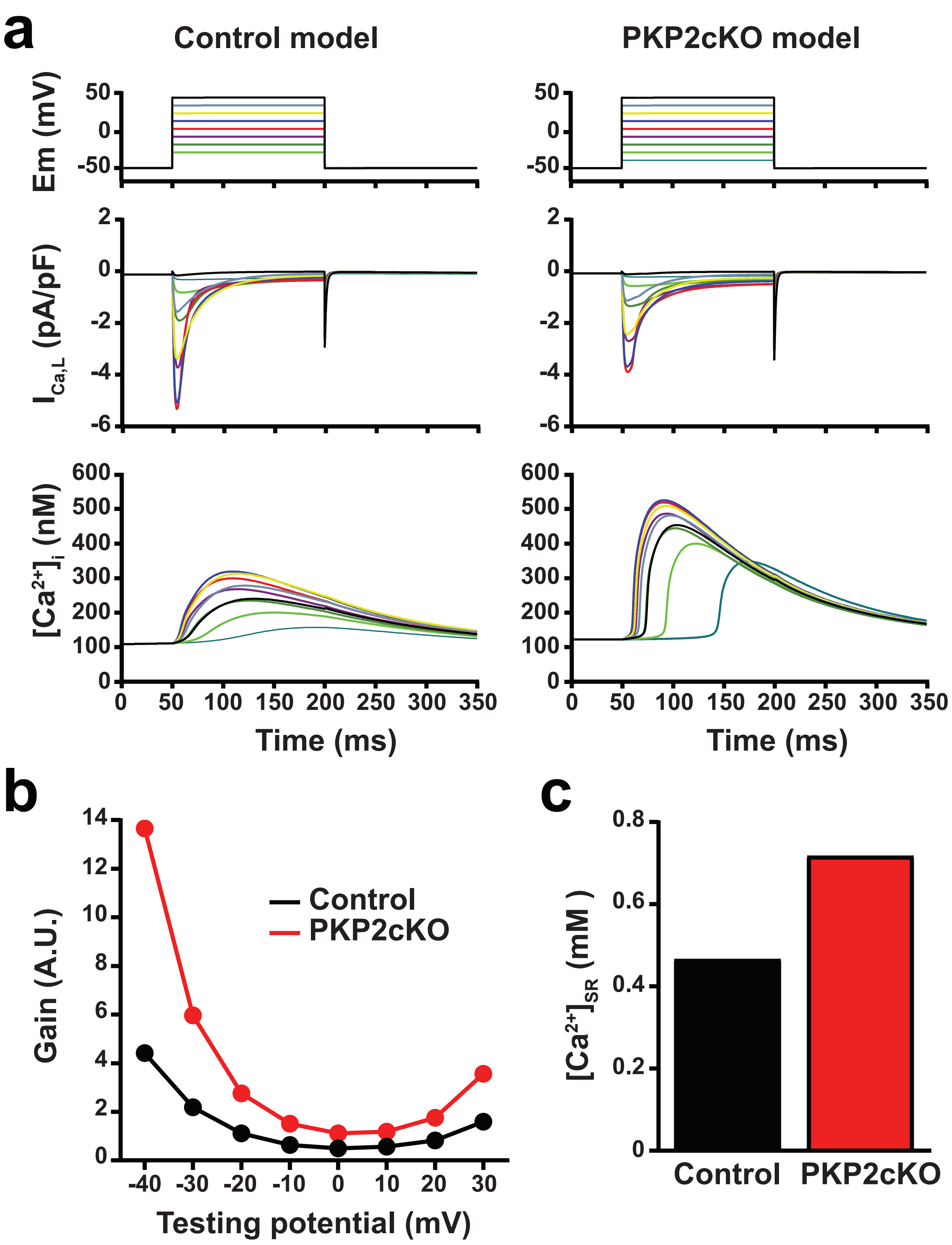

Supplementary Figure 8: Mathematical simulations of e-c coupling gain factor. (**a**) Traces obtained using the original model (left), or one in which we modified the amplitude and kinetics of the calcium current, as well as the abundance of RyR2 and Casq2 as per the experimental data (see also "Methods"). An increase in e-c coupling gain (**b**) and in free calcium SR load (**c**) were observed in the PKP2-cKO model.

Supplementary Fig. 8

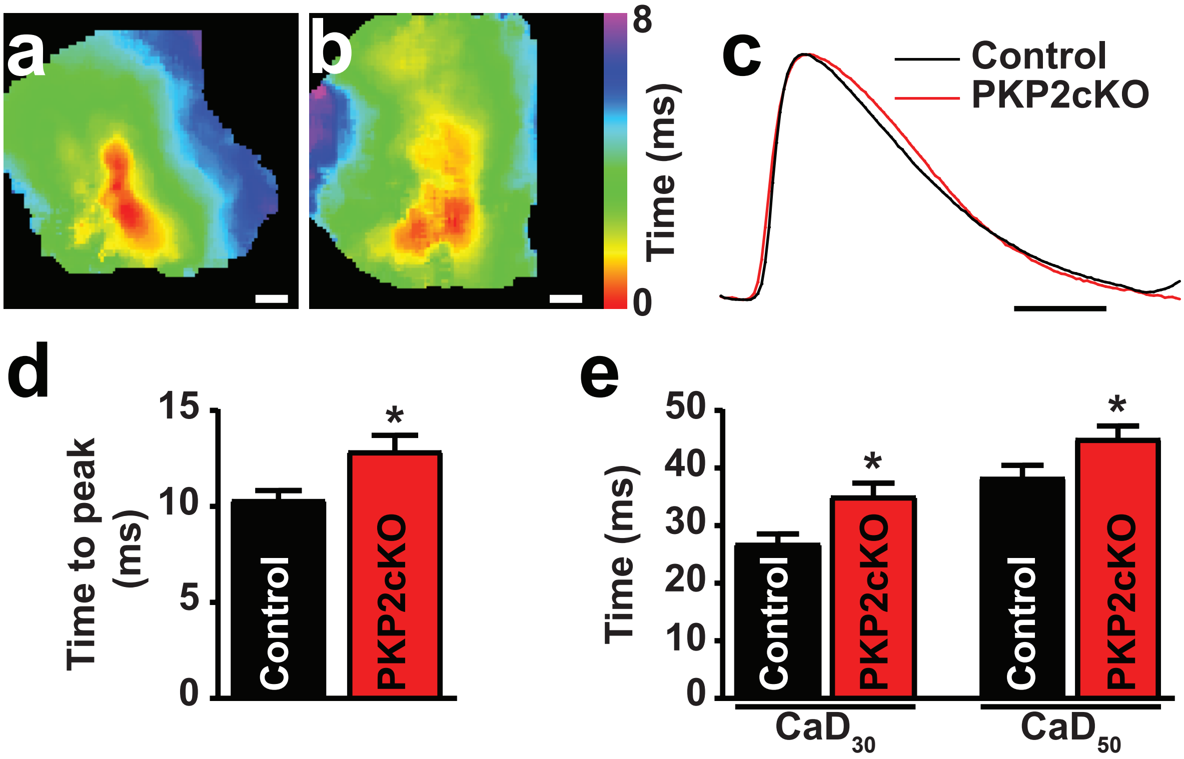

Supplementary Figure 9: Calcium mapping in isolated hearts. (**a**) and (**b**) Activation maps showing calcium transients measured at 120 msec BCL in control and PKP2-cKO respectively. Scale bar: 1 mm. (**c**) Average calcium activation transients for the maps shown in a and b, normalized to peak amplitude. Scale bar: 25 msec. (**d**) Average time-to-peak of the calcium transients in controls (black bars, n=6) and PKP2-cKO (red bars, n=6) * $p<0.05$. (**e**) Average calcium transient duration from dF/dt Max to 30% and 50% of cytosolic extrusion in controls (black bars, n=6) and PKP2-cKO (red bars, n=6) * $p<0.05$.

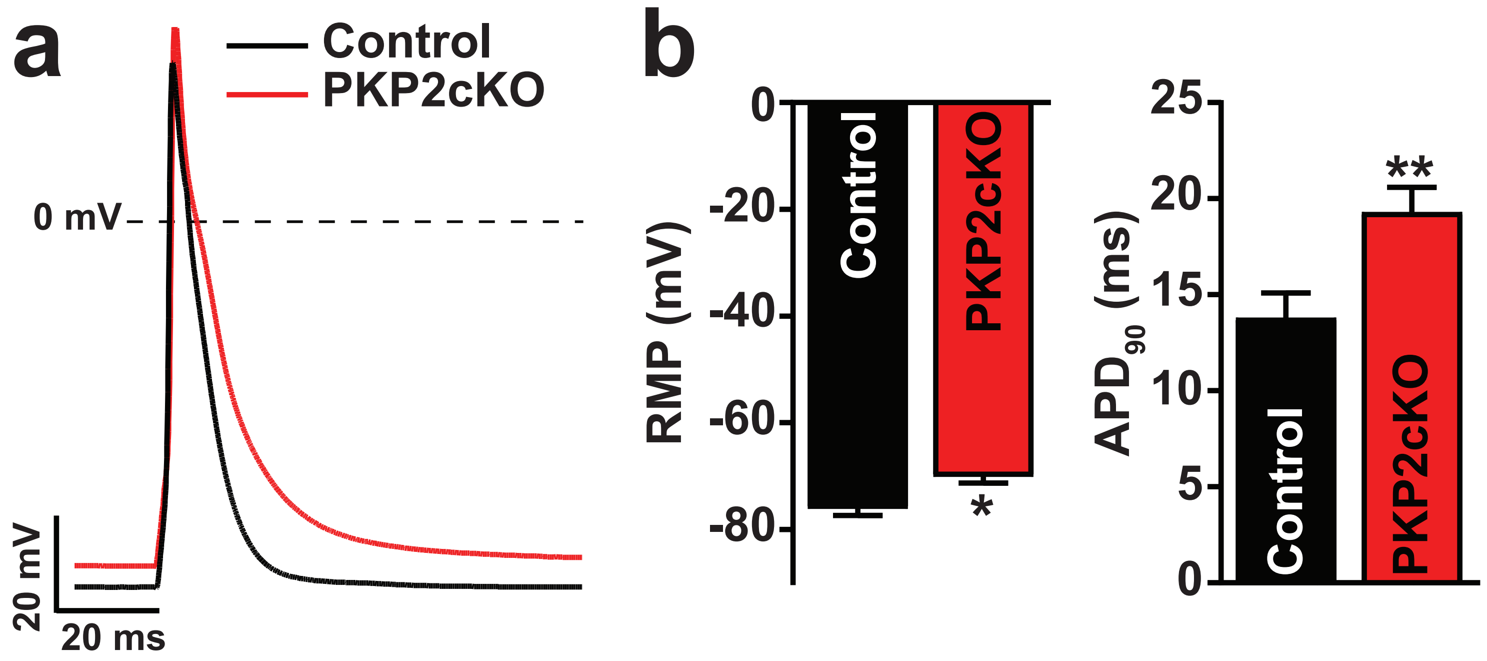

Supplementary Figure 10: Action potential of ventricular cardiomyocytes. (**a**) Action potentials recorded in Control (black) and PKP2-cKO (21dpi, red) isolated cardiomyocytes. (**b**) Bar graphs depicting resting membrane potential (RMP) and action potential duration at 90% of repolarization (APD_{90}) in Control (n=10; 2 mice) and PKP2-cKO (n=14; 3 mice) cardiomyocytes. * p<0.01, ** p<0.001 versus Control.

Supplementary Fig. 10

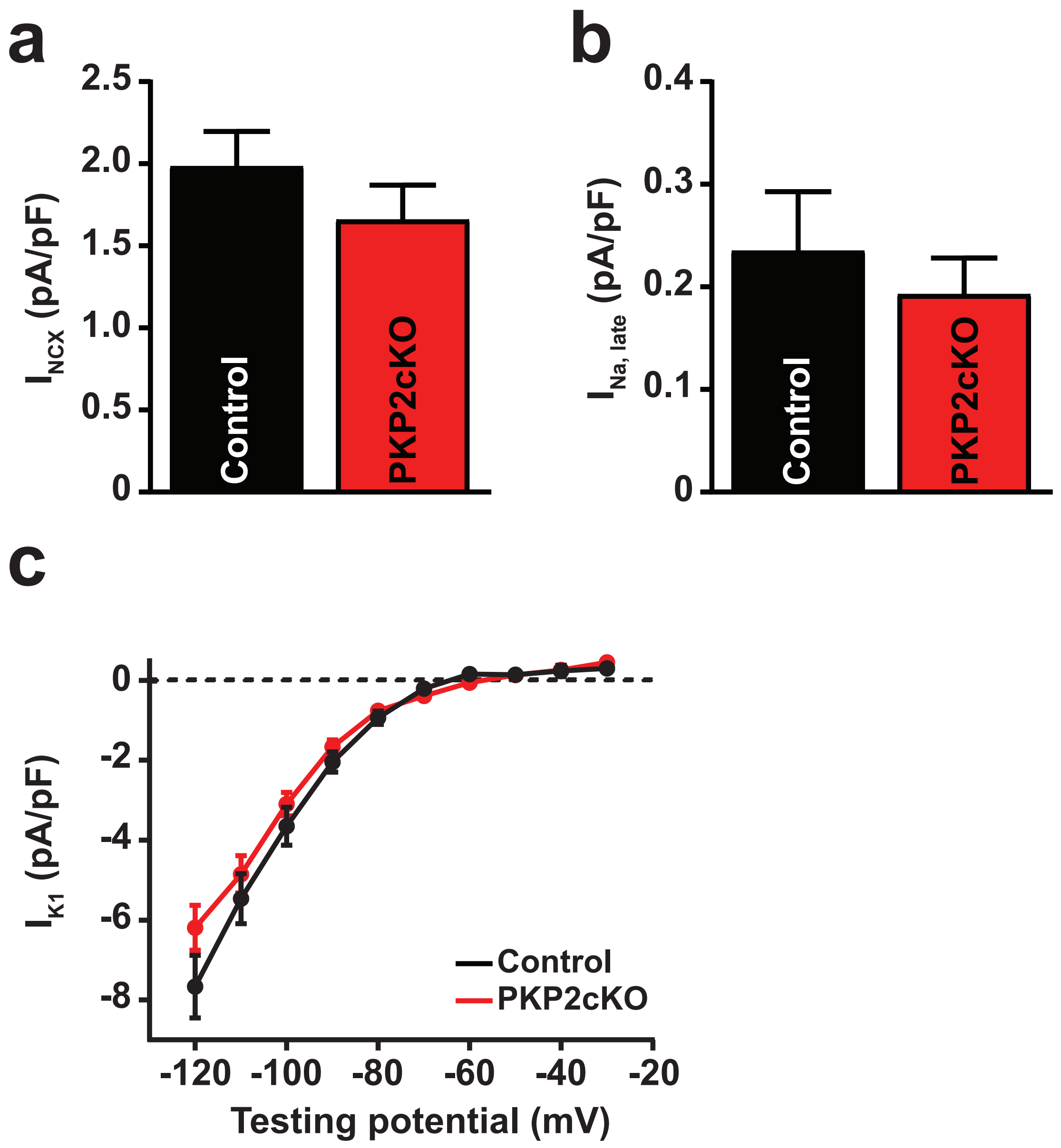

Supplementary Figure 11: (**a**) Quantification of Ni^{2+}-sensitive NCX current in Control (black; n=10; 2 mice) and 21 dpi PKP2-cKO (21 dpi, red; n=10; 2 mice) cardiomyocytes (**b**) Quantification of TTX-S late sodium current in Control (black; n=10; 2 mice) and 21 dpi PKP2-cKO (21dpi , red; n=9; 2 mice) cardiomyocytes. (**c**) Quantification of inward-rectifier I_{K1} current in control (black; n=10; 2 mice) and 21 dpi PKP2-cKO (red; n=12; 2 mice) cardiomyocytes. None of the comparisons yielded statistical significance by Student's unpaired t test

Supplementary Fig. 11

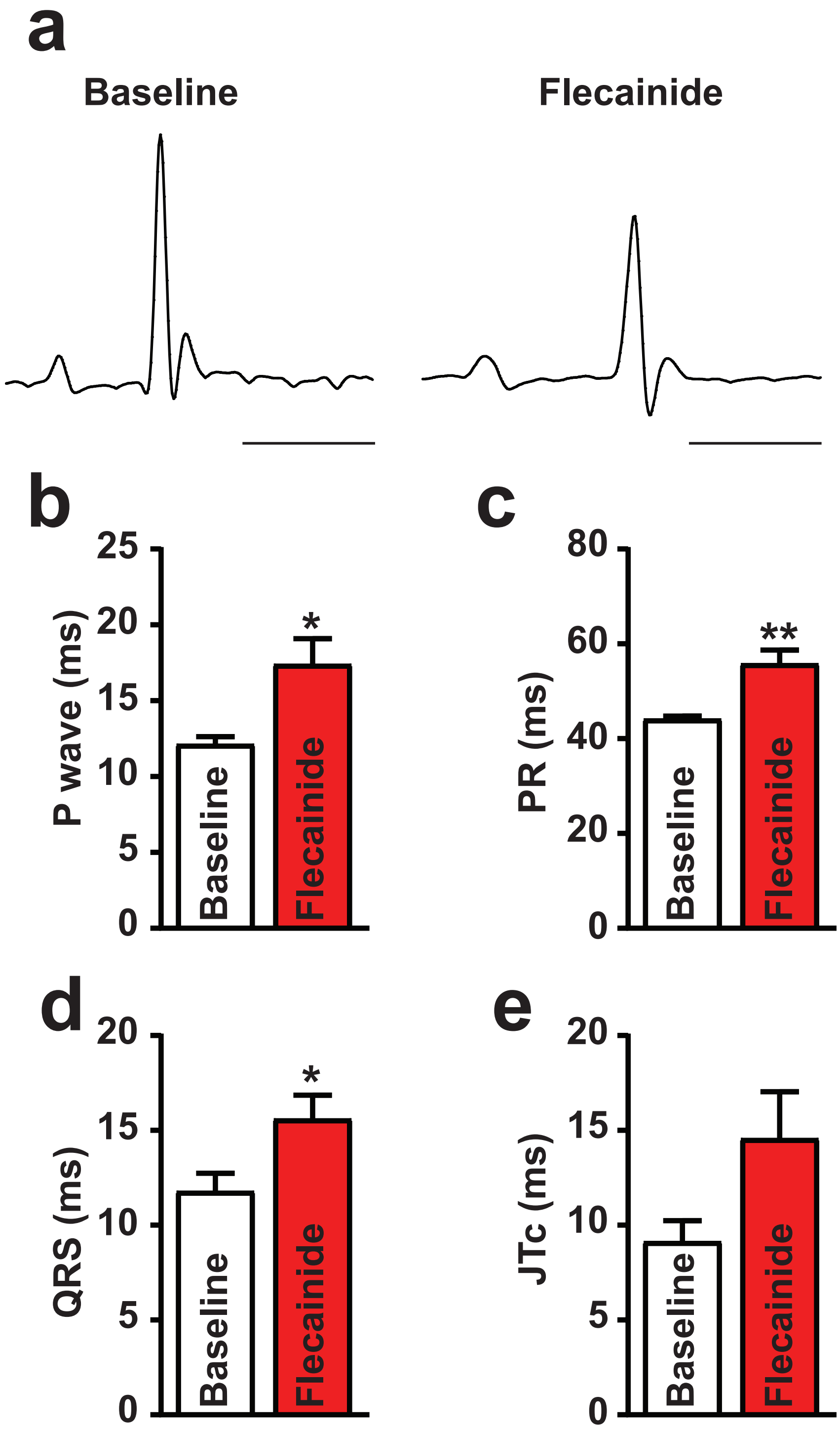

Supplementary Figure 12: ECG characteristics of PKP2-cKO mice after flecainide challenge at 21 dpi (**a**) Lead II ECGs of PKP2-cKO mice before and 15 minutes after flecainide 40 mg/kg i.p. challenge (n=6). Scale = 100 ms. (**b-e**). Bar graphs depicting P wave, PR interval, QRS interval and JTc interval durations before (white bars) and after (red bars) flecainide injection challenge. Drug effect is demonstrated by the expected increase in P, PR and QRS, but not JT intervals duration. Student's paired t-test, * p<0.01, ** p<0.001.

Supplementary Fig. 12

Supplementary Table 1: Echocardiographic parameters in control and PKP2-cKO mice. Bold numbers indicate significant differences versus baseline by Student's paired T test.

	Controls						PKP2cKO					
	**Baseline**	**14 dpi**	**21 dpi**	**28 dpi**	**35 dpi**	**42 dpi**	**Baseline**	**14 dpi**	**21 dpi**	**28 dpi**	**35 dpi**	**42 dpi**
LVEF (%)	52.19 ± 0.93 (15)	51.95 ± 1.33 (18)	54.93 ±1.32 (13)	48.25 ± 2.54 (4)	53.45 ± 1.48 (7)	51.06 ± 1.22 (10)	52.48 ± 0.86 (21)	48.60 ± 1.03 (13)	**43.71 ± 1.35 (19) *****	**30.31 ± 2.25 (16) *****	**21.84 ± 1.81 (15) *****	**20.43 ± 1.90 (10) *****
FS (%)	33.74 ± 1.40 (15)	31.43 ± 0.94 (18)	31.36 ± 1.85 (13)	31.19 ± 3.16 (4)	30.34 ± 1.76 (7)	32.76 ± 1.49 (10)	30.67 ± 0.68 (22)	28.29 ± 0.95 (13)	**24.48 ± 1.08 (25) *****	**17.87 ± 1.09 (16) *****	**13.79 ± 0.91 (14) *****	**15.11 ± 1.23 (10) *****
LVEDV (mm^3)	62.91 ± 2.26 (15)	64.91 ± 2.10 (18)	64.16 ± 2.55 (13)	62.34 ± 3.22 (4)	64.28 ± 4.71 (7)	63.82 ± 1.95 (10)	69.69 ± 2.71 (22)	71.78 ± 4.51 (13)	76.47 ± 4.51 (19)	**78.85 ± 4.85 (16) ***	**90.26 ± 6.98 (14) ****	**103.17 ± 9.08 (10) ****
LVESV (mm^3)	29.18 ± 1.08 (15)	31.19 ± 1.27 (18)	28.70 ± 1.13 (13)	32.10 ± 1.50 (4)	29.67 ± 2.57 (7)	31.25 ± 1.43 (10)	32.52 ± 1.41 (22)	35.47 ± 2.60 (13)	**43.39 ± 3.06 (19) *****	**55.05 ± 4.00 (16) *****	**70.43 ± 6.08 (14) *****	**82.12 ± 7.51 (10) *****
RVA (mm^2)	1.98 ± 0.15 (7)	2.31 ± 0.13 (8)	2.19 ± 0.11 (5)	2.47 ± 0.36 (3)	2.62 ± 0.16 (6)	2.30 ± 0.11 (7)	2.07 ± 0.14 (21)	**2.54 ± 0.10 (12)***	**3.14 ± 0.17 (18) *****	**3.95 ± 0.21 (16) *****	**4.95 ± 0.40 (11) *****	**5.03 ± 0.38 (9) *****
RV_VTI (mm/s)	25.89 ± 1.50 (14)	27.02 ± 0.96 (18)	28.38 ± 0.82 (13)	25.23 ± 2.40 (4)	26.94 ± 1.81 (7)	26.28 ± 1.27 (10)	27.05 ± 1.09 (22)	26.69 ± 2.03 (12)	25.99 ± 1.06 (19)	**17.37 ± 0.85 (16) *****	**13.82 ± 0.62 (12) *****	**15.42 ± 1.20 (9) ****
PVA (mm^2)	1.37 ± 0.03 (14)	1.45 ± 0.04 (18)	1.38 ± 0.04 (13)	1.32 ± 0.13 (4)	1.42 ± 0.10 (7)	1.40 ± 0.05 (10)	1.29 ± 0.03 (22)	**1.48 ± 0.04 (13) *****	**1.55 ± 0.04 (19) *****	**1.50 ± 0.06 (16) ***	**1.73 ± 0.10 (12) *****	**1.75 ± 0.12 (10) *****

*LVEF: Left Ventricular Ejection Fraction; FS: Fraction Shortening; LVEDV: Left Ventricular End-Diastolic Volume; LVESV: Left Ventricular End-Systolic Volume; RVA: Right Ventricular Area; VTI: Velocity Time Integral of the Right Ventricular Outflow Tract; PVA: Pulmonary Vein Area. Paired t-test *p<0.05, **p<0.01, ***p<0.001 versus Baseline.*

Supplementary Table 2: Antibodies used in this study listed by manufacturer, animal source, dilution and mode of use.

	Reference	Manufacturer	Class	Host	Working concentration	Application
**PKP2**	K44262M	Biodesign International, Meridian Life Sciences	monoclonal	mouse	1:100	WB
**AnkyrinB**	N105-17	BioLegend	monoclonal	mouse	1:100	WB/IHC
**Cav1.2**	ACC-003	Alomone	polyclonal	rabbit	1:500	WB
**Casq2**	PA1-913	ThermoFisher	polyclonal	rabbit	1:1000<br>1:200	WB<br>IHC
**RyR2**	MA3-916	ThermoFisher	monoclonal	mouse	1:2000	WB
**RyR2 p-S2808**	A010-30	Badrilla	polyclonal	rabbit	1:5000	WB
**RyR2 p-S2814**	A010-31	Badrilla	polyclonal	rabbit	1:5000	WB
**TriadinT32**	MA3-927	ThermoFisher	monoclonal	mouse	1:400	WB
**NCX**	sc-32881	SantCruz	polyclonal	rabbit	1:500	WB
**SERCA2**	PA5-29380	ThermoFisher	polyclonal	rabbit	1:500	WB
**Phospholamban**	sc-393990	SantCruz	monoclonal	mouse	1:1000	WB
**Phospholamban p-S16**	sc-12963	SantCruz	polyclonal	rabbit	1:1000	WB
**Phospholamban p-T17**	sc-17024	SantCruz	polyclonal	rabbit	1:1000	WB
**Cx43**	Clone 4E6.2<br>Ab1728	Millipore<br>Millipore	monoclonal<br>polyclonal	mouse<br>rabbit	1:500<br>1:200	WB<br>IHC
**β-catenin**	C2206	Sigma	polyclonal	rabbit	1:100	IHC
**JPH2**	JPH2	SantCruz	monoclonal	mouse	1:500	WB
**BIN-1**	Clone 99D	Sigma	monoclonal	mouse	1:50	IHC
**PKC**	#2056	Cell Signaling	polyclonal	rabbit	1:500	WB
**p-PKC**	#9375	Cell Signaling	polyclonal	rabbit	1:500	WB
**CaMKII**	PA5-22168	ThermoFisher	polyclonal	rabbit	1:500	WB
**CaMKII p-T286**	MA1-047	ThermoFisher	monoclonal	mouse	1:250	WB
**Dsc-2**	ab72792	Abcam	polyclonal	rabbit	1:250	WB
**Nav1.5**	S0819	Sigma	polyclonal	rabbit	1:500	WB
**GAPDH**	G109A	Fitzerald	monoclonal	mouse	1:15000	WB
**IRDye 800CW**	P/N 926-32210	LI-COR	polyclonal	goat anti-mouse	1:15000	WB
**IRDye 680RD**	P/N 926-68071	LI-COR	polyclonal	goat anti-rabbit	1:15000	WB
**Alexa Fluor488**	A-11008	ThermoFisher	polyclonal	goat anti-rabbit	1:400	IHC
**Alexa Fluor568**	A-11004	ThermoFisher	polyclonal	goat anti-mouse	1:400	IHC
**Alexa Fluor647**	A-21235	ThermoFisher	polyclonal	goat anti-mouse	1:400	IHC

Supplementary Table 3: Minimal T-tubule-junctional SR distance (nm) in controls and PKP2-cKO at 21 dpi.

	Average minimal distance (nm)	p-value
**Control (n=30)**	11.08 ± 0.98	
**PKP2cKO_1 (n=41)**	11.48 ± 0.74	0.74
**PKP2cKO_2 (n=41)**	14.91 ± 2.15	0.15

Supplementary Table 4: Ryanodine binding assays in control (n=6) and PKP2-cKO at 21 dpi (n=6) cardiomyocytes

Parameter	Control (n=6)	PKP2cKO (n=6)	p-value
RyR2 expression normalized to GAPDH (a.u.)	1.00 ± 0.08	0.66 ± 0.11	0.0347
Maximum [^{3}H]Ryanodine binding corrected for expression (fmol/mg of protein)	29.33 ± 3.27	31.62 ± 4.11	0.6729
[Ca^{2+}] at 50% RyR2 activation, EC_{50} (μM)	1.36 ± 0.28	0.94 ± 0.19	0.2443
Maximum [^{3}H]Ryanodine binding (fmol/mg of protein)	29.42 ± 4.13	19.62 ± 2.90	0.0841
